# Polycomb Associates Genome-wide with a Specific RNA Polymerase II Variant, and Regulates Metabolic Genes in ESCs

**DOI:** 10.1016/j.stem.2011.12.017

**Published:** 2012-02-03

**Authors:** Emily Brookes, Inês de Santiago, Daniel Hebenstreit, Kelly J. Morris, Tom Carroll, Sheila Q. Xie, Julie K. Stock, Martin Heidemann, Dirk Eick, Naohito Nozaki, Hiroshi Kimura, Jiannis Ragoussis, Sarah A. Teichmann, Ana Pombo

**Affiliations:** 1 Genome Function Group, MRC Clinical Sciences Centre, Imperial College School of Medicine, Hammersmith Hospital Campus, Du Cane Road, London W12 0NN, UK; 2 Gulbenkian Institute of Science, R. Quinta Grande, 6-2780 Oeiras, Portugal; 3 Computational Genomics Group, MRC Laboratory of Molecular Biology, Hills Road, Cambridge CB2 2QH, UK; 4 Department of Molecular Epigenetics, Helmholtz Center Munich, Center of Integrated Protein Science, 81377 Munich, Germany; 5 Bio-Frontier Research Center, Tokyo Institute of Technology, Yokohama 226-8503, Japan; 6 Graduate School of Frontier Biosciences, Osaka University, Suita, Osaka 565-0871, Japan; 7 Genomics Research Group, Wellcome Trust Centre for Human Genetics, 7 Roosevelt Drive, Oxford OX3 7BN, UK

## Abstract

Polycomb repressor complexes (PRCs) are important chromatin modifiers fundamentally implicated in pluripotency and cancer. Polycomb silencing in embryonic stem cells (ESCs) can be accompanied by active chromatin and primed RNA polymerase II (RNAPII), but the relationship between PRCs and RNAPII remains unclear genome-wide. We mapped PRC repression markers and four RNAPII states in ESCs using ChIP-seq, and found that PRC targets exhibit a range of RNAPII variants. First, developmental PRC targets are bound by unproductive RNAPII (S5p^+^S7p^−^S2p^−^) genome-wide. Sequential ChIP, Ring1B depletion, and genome-wide correlations show that PRCs and RNAPII-S5p physically bind to the same chromatin and functionally synergize. Second, we identify a cohort of genes marked by PRC and elongating RNAPII (S5p^+^S7p^+^S2p^+^); they produce mRNA and protein, and their expression increases upon PRC1 knockdown. We show that this group of PRC targets switches between active and PRC-repressed states within the ESC population, and that many have roles in metabolism.

## Graphical abstract

**Graphical Abstract:**

**Figure:**
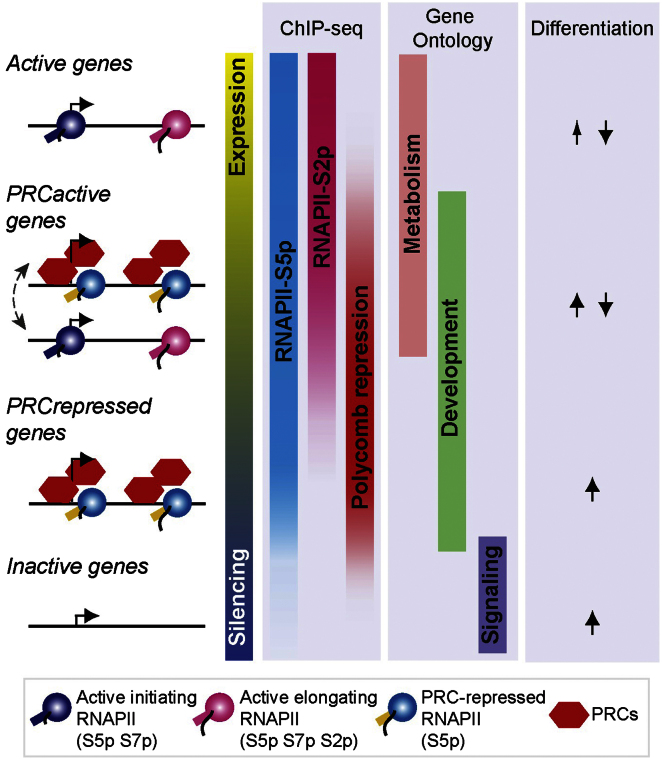

**Highlights:**

► A unique RNAPII variant (S5p^+^S7p^−^S2p^−^) binds PRC targets genome-wide in ESCs ► RNAPII-S5p and PRC coincide in time and localization, and show proportional abundance ► Novel, active PRC-target genes identified in ESCs include metabolic genes ► Active PRC targets switch between on/off (active/PRC) states in the ESC population

## Introduction

ESCs are characterized by their abilities to self-renew and differentiate into all somatic cell types (Jaenisch and Young, 2008), but the molecular mechanisms underlying pluripotency are not fully understood. Pluripotency depends on the silencing of developmental regulator genes by two major PRCs that modify histones (Richly et al., 2010; Schwartz and Pirrotta, 2008). PRC1 monoubiquitinylates H2AK119 (H2Aub1) via the ubiquitin ligase Ring1B. PRC2 catalyzes dimethylation and trimethylation of H3K27 (H3K27me2/3) via its histone methyltransferase (HMT) Ezh2. In mammals, PRC2-mediated H3K27me3 at repressed genes can be accompanied by markers of gene activity: (1) histone marks characteristic of active genes, such as H3K4me3, that generate bivalent chromatin domains, (2) the binding of RNAPII and transcription factors, and (3) transcription (Azuara et al., 2006; Bernstein et al., 2006; Brookes and Pombo, 2009; Enderle et al., 2011; Schwartz and Pirrotta, 2008). PRC repression mechanisms in the context of gene activity are not clear.

RNAPII activity is regulated by complex phosphorylation of the C-terminal domain (CTD) of its largest subunit, which comprises 52 repeats of the heptapeptide sequence Y1-S2-P3-T4-S5-P6-S7. CTD modifications during the active transcription cycle recruit specific histone modifiers and RNA processing factors, promoting active chromatin and appropriate RNA maturation (Brookes and Pombo, 2009; Weake and Workman, 2010). S5 phosphorylation (S5p) correlates with initiation, capping, and H3K4 HMT recruitment. S2 phosphorylation (S2p) correlates with elongation, splicing, polyadenylation, and H3K36 HMT recruitment. S7 phosphorylation (S7p) is present at promoter and coding regions of active genes in mammalian cells (Chapman et al., 2007), and is thought to occur together with S5p and S2p (Akhtar et al., 2009; Tietjen et al., 2010). Studies of RNAPII modification at PRC-target genes in ESCs have been limited. High levels of RNAPII-S5p were detected at promoter and coding regions of nine PRC targets in the absence of S2p (Stock et al., 2007). However, probing with antibody 8WG16 against hypophosphorylated CTD detects little or no RNAPII at PRC-target genes (Guenther et al., 2007; Stock et al., 2007).

The presence of PRCs, RNAPII-S5p, and repressive/active histone marks at PRC targets in ESCs has been seen after population-based ChIP assays (Alder et al., 2010; Bernstein et al., 2006; Mikkelsen et al., 2007; Stock et al., 2007). However, true colocalization of opposing histone modifications has been observed by sequential ChIP for very few genes, raising questions about the significance of chromatin bivalency genome-wide (De Gobbi et al., 2011).

**Figure 1:**
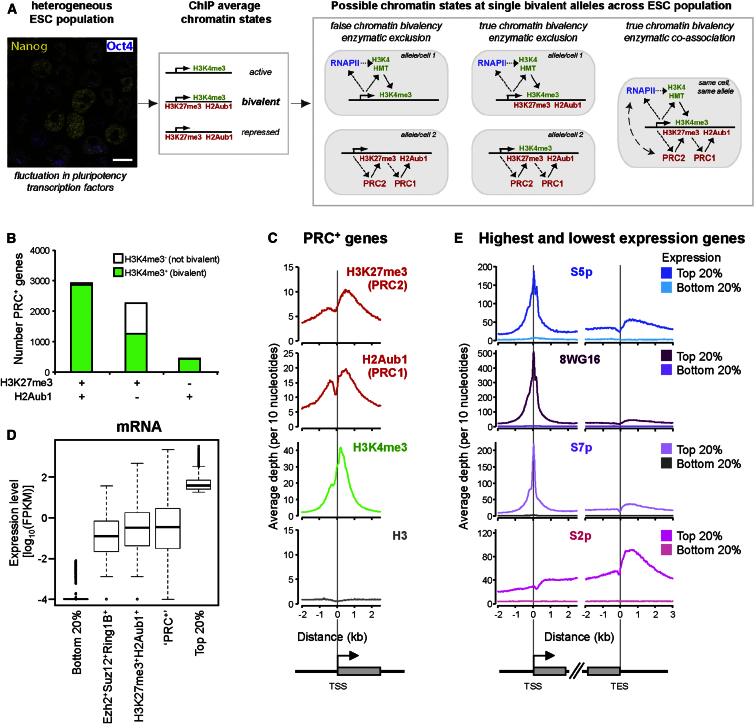
Mapping PRCs and RNAPII to Investigate Chromatin Bivalency in ESCs (A) ESCs are naturally heterogeneous for expression of some transcription factors, including Nanog and Oct4 (yellow and blue, respectively; left panel, whole-cell immunofluorescence; bar: 10 μm). Detection of H3K4me3 and H3K27me3/H2Aub1 at the same chromatin using population-based ChIP (central panel) may reflect true colocalization of the modifications, or may be due to dynamically or spatially separated marks arising from ESC interconversion (right panel). Furthermore, chromatin bivalency may occur with or without physical association of responsible enzymatic activities, due to greater longevity of histone modifications. Dotted arrow, recruitment; solid arrow, enzymatic modification. (B) Genes associated with both H3K27me3 and H2Aub1, or with H2Aub1 alone, are predominantly occupied by H3K4me3 (98% and 96%, respectively). Only 56% of H3K27me3-only genes are bound by H3K4me3. (C) Average ChIP-seq profiles of histone modifications at PRC^+^ genes (H3K27me3^+^ and/or H2Aub1^+^). (D) mRNA-seq expression levels for the 20% most highly and 20% least expressed genes, and for PRC-target genes marked by Ezh2, Suz12, and Ring1B, by both H3K27me3 and H2Aub1, and by H3K27me3 and/or H2Aub1 (PRC^+^). PRC targets show a wide range of expression levels. (E) Average ChIP-seq profiles of RNAPII for the 20% of genes with highest (bright colors) and lowest (pale colors) expression levels. See also Figures S1 and S2.

Furthermore, it is widely accepted that all ESC cultures exhibit functional heterogeneity, expressing variable levels of pluripotency transcription factors (Figure 1A), which may influence their propensity to differentiate into specific lineages upon appropriate signals (Carter et al., 2008; Graf and Stadtfeld, 2008). Under self-renewing conditions, ESCs interconvert between these states (Canham et al., 2010; Singh et al., 2007), reminiscent of the early stages of blastocyst differentiation. Important transcription factors showing cell-to-cell fluctuations include Nanog (Chambers et al., 2007; Singh et al., 2007), Rex1 (Toyooka et al., 2008), and Stella (Hayashi et al., 2008). It is therefore debated whether chromatin bivalency could be explained by chromatin state switching due, at least in part, to ESC heterogeneity (Figure 1A). It also remains unclear whether true coassociation of bivalent histone modifications reflects simultaneous binding of PRCs and RNAPII, known to coordinate deposition of H3K27me3 and H3K4me3, respectively, due to the greater longevity of histone modifications. We set out to explore these phenomena. We identify different classes of PRC-target genes that exhibit distinct RNAPII variants and expression levels and explore their regulation.

## Results

### Chromatin Bivalency Revisited

To further investigate chromatin bivalency in ESCs, we produced genome-wide data sets for markers of Polycomb repression and transcriptional activation, and reanalyzed published data sets (Table S1 available online). Our understanding of bivalency has relied on mapping of H3K27me3 and H3K4me3 (Azuara et al., 2006; Bernstein et al., 2006; Mikkelsen et al., 2007), but H3K27me3 represents only the activity of PRC2, and not that of PRC1. PRC1 catalyzes H2Aub1 deposition, but there is currently no genome-wide H2Aub1 data set available in mouse ESCs. Mapping of PRC1-component Ring1B identified PRC2 binding in the absence of PRC1 (Ku et al., 2008).

We performed ChIP-seq for H2Aub1 and mapped PRC1 catalytic subunit Ring1B to increase signal depth. We also remapped high-quality, publicly available ChIP-seq data for PRC2 subunits Ezh2 and Suz12, and PRC2 histone modification H3K27me3. We performed ChIP-seq for four RNAPII states (S5p, S2p, S7p, 8WG16) and for H3K36me3, and remapped published H3K4me3 data, using these as markers of transcriptional activity.

We reexamined the extent of chromatin bivalency by considering both H3K27me3 and H2Aub1, and classifying PRC-positive genes (PRC^+^) according to their association with H3K27me3 and/or H2Aub1 (Table S2). Genes were classified by integrating levels of ChIP enrichment within windows of interest (Hebenstreit et al., 2011). In contrast with classifications based on the presence of PRC enzymatic subunits, mapped PRC-instigated histone modifications constitute a functional readout of PRC repression. This takes into account, for example, that Ring1B is present in complexes other than PRC1 (Sánchez et al., 2007), and that Ezh1 can compensate for Ezh2 in PRC2 (Shen et al., 2008).

We identified a large cohort of PRC^+^ genes (n = 5,628) that are associated with both H2Aub1 and H3K27me3 (n = 2,931), associated with only H3K27me3 (n = 2,254), or associated with only H2Aub1 (n = 443; Figure 1B). We validated this result using a recent higher-depth H3K27me3 data set (Lienert et al., 2011; Figure S1A). ChIP-seq signal enrichment at TSSs correlates well between Mikkelsen and Lienert data sets (Figure S1B). High numbers of H3K27me3^+^ genes (n = 5,571) were also recently identified using independent H3K27me3 ChIP-seq data and a different classification strategy (Young et al., 2011).

Comparisons between H3K27me3, H2Aub1, and H3K4me3 presence show that H2Aub1^+^ is more closely associated with chromatin bivalency than H3K27me3 (Figure 1B). The vast majority (97%) of H2Aub1^+^ genes are bivalent (i.e. also occupied by H3K4me3), irrespective of H3K27me3, whereas only 79% of H3K27me3^+^ genes are H3K4me3^+^. Analysis of the alternative H3K27me3 data set (Lienert et al., 2011) confirms this result (Figure S1A).

Our analyses are consistent with previous studies suggesting that PRC2 can bind independently of PRC1 (Ku et al., 2008). The newly classified PRC^+^ genes, based on their association with H2Aub1 and/or H3K27me3, are also associated with the catalytic subunits responsible for these histone modifications, Ezh2 and Ring1B, as anticipated (Figure S1C).

Mapping average ChIP-seq profiles of H3K27me3, H2Aub1, H3K4me3, and core H3 at transcription start sites (TSSs) of PRC^+^ genes demonstrates broad peaks of PRC-instigated H3K27me3 and H2Aub1 enrichment, together with a tighter peak of H3K4me3 (Figure 1C). Core H3 is not enriched at the TSS (Figure 1C) and so cannot explain the high TSS levels of H3 modifications observed. Catalytic PRC subunits (Ezh2 and Ring1B) show similar distributions to the marks they deposit (Figure S1C).

### PRC-Target Genes Are Not Universally Silent, with Some Exhibiting Intermediate or High Expression Levels

To explore the functional significance of chromatin bivalency, we determined the expression levels of PRC^+^ genes after mapping mRNA by high-throughput sequencing (mRNA-seq). Surprisingly, the PRC^+^ cohort contains not only silent genes, but also genes with intermediate and high expression (Figure 1D). This holds true for genes bound by both H3K27me3 and H2Aub1, and also for PRC-target genes defined by their association with PRC subunits (Ezh2, Suz12, Ring1B; Figure 1D). The large range of expression levels at PRC^+^ genes is also seen using published mRNA data sets (Cloonan et al., 2008; Mikkelsen et al., 2007) determined with different methodologies and ESC lines (Figure S1D).

The range of expression levels at PRC targets suggests that PRCs do not act as absolute silencers, but may regulate the extent of RNAPII transcriptional activity, as described in *Drosophila* (Enderle et al., 2011; Schwartz and Pirrotta, 2008). Although mammalian PRCs are known to exert a repressive effect, substantial expression has been previously seen at PRC2-target genes (Nishiyama et al., 2009; Young et al., 2011).

### RNAPII Modification Genome-wide at Active and Silent Genes in ESCs

To explore the range of expression states at PRC targets, we mapped RNAPII presence and modification across the ESC genome. We first validated our RNAPII modification data sets by aligning ChIP-seq data relative to TSSs and transcription end sites (TESs) of the 20% of genes with the highest or lowest expression levels in the genome (3,772 genes/group; Figure 1E). The 20% least expressed genes show little or no signal for RNAPII, PRCs, or mapped histone marks (Figure 1E, Figure S1E), demonstrating that ESCs harbor a large group of genes silent in the absence of RNAPII or PRC marks.

Genes with the highest expression are associated with all RNAPII marks (Figure 1E), H3K4me3, and H3K36me3, but not Polycomb (H3K27me3, Ezh2, Suz12, H2Aub1, and Ring1B), as expected (Figure S1E). At these genes, RNAPII-S5p, 8WG16, S7p, and H3K4me3 peak at promoters, which is consistent with RNAPII promoter-proximal pausing at active genes (Core and Lis, 2008). RNAPII-S5p is detected at low levels throughout coding regions and shows a small increase downstream of TESs. S2p and H3K36me3 increase through coding regions; S2p peaks ∼700 bp downstream of TESs. RNAPII accumulation beyond TESs, marked by both S2p and S5p, may reflect termination and S2p-dependent coordination of mRNA polyadenylation. Inspection of ChIP-seq profiles across single genes confirms these average active and silent configurations (Figure S2A).

### Genome-wide RNAPII Modification at PRC Targets in ESCs

We next explored the RNAPII state at PRC^+^ genes. First, we inspected ChIP-seq profiles at single PRC^+^ genes with different expression levels and found distinct RNAPII profiles (Figure S2B). Silent PRC-target genes were generally associated with S5p only, whereas actively expressed PRC targets were occupied by both S5p and S2p. Heatmaps representing RNAPII modification at PRC^−^ and PRC^+^ genes, ordered according to mRNA expression levels, show that expression is positively associated with 8WG16, S7p, and S2p within both groups. The heatmaps also show genome-wide association between PRCs and RNAPII-S5p (Figure S2C).

To investigate chromatin bivalency and interplay between PRCs and RNAPII, we first looked genome-wide at the association of PRCs with the unusual RNAPII variant (S5p^+^S2p^−^8WG16^−^) previously identified in a panel of PRC-target developmental genes (Stock et al., 2007).

**Figure 2:**
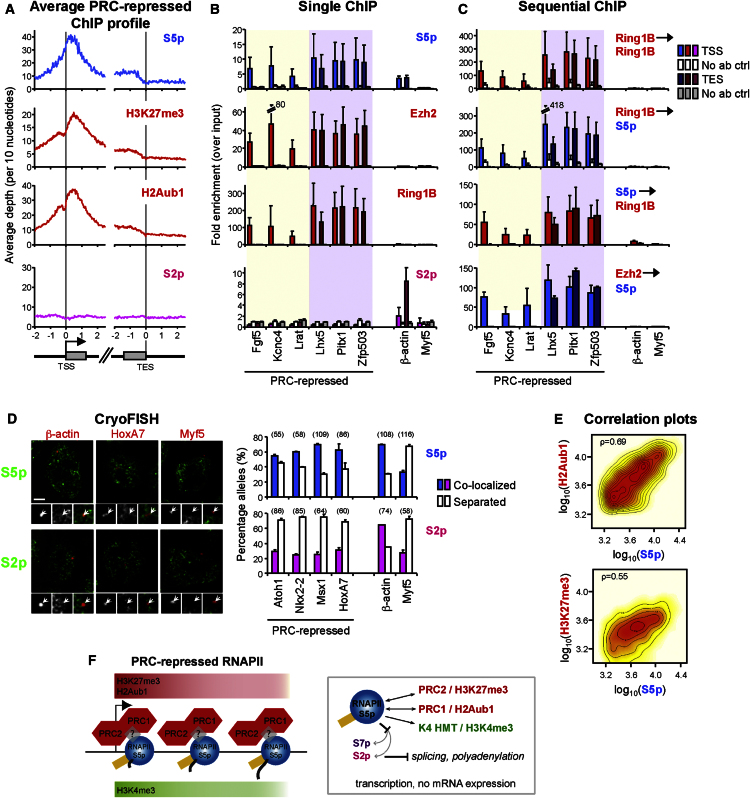
RNAPII-S5p Coassociates with PRC1 and PRC2 through Coding Regions of PRC-Repressed Genes (A) Average ChIP-seq profiles at PRC-repressed genes (H3K27me3^+^H2Aub1^+^) associated with RNAPII-S5p^+^S2p^−^8WG16^−^. S5p, H3K27me3, and H2Aub1 have similar broad profiles at TSSs and through coding regions. (B) Occupancy of RNAPII-S5p, Ezh2, Ring1B, and S2p was confirmed by ChIP-qPCR at TSSs (light) and TESs (dark) of *Active* (*β-actin*), *Inactive* (*Myf5*), and PRC-repressed genes with or without detectable TES S5p enrichment. Background levels (mean enrichment from control antibodies and beads alone) at TSSs (white bars) and TESs (gray bars) are shown. Mean and standard deviations (SD) from three to four biological replicates are shown. (C) Sequential ChIP shows RNAPII-S5p coassociation with Ring1B and Ezh2 at PRC-repressed genes. Background levels (white or gray bars) represent mean enrichment after first ChIP with Ring1B followed by re-ChIP with no antibody. No DNA was recovered from S5p→mock or Ezh2→mock (control bars are not shown for S5p→Ring1B or Ezh2→S5p). Mean and SD from four to six biological replicates are shown. (D) PRC-repressed genes associate with S5p at a similar frequency to that of active gene *β-actin*, but not with S2p. Localization by immuno-cryoFISH of PRC-repressed or control loci (red, arrows) relative to S5p and S2p sites (green) in ESCs was scored as “colocalized” (>1 pixel overlap) or “separate.” Bar: 2 μm. Number of loci analyzed are shown in brackets. (E) Positive correlation between S5p and H2Aub1 or H3K27me3 levels in 2kb TSS windows of PRC-repressed genes (Spearman's rank correlation coefficient; ρ) are shown. (F) PRC-repressed genes are associated simultaneously with nonproductive RNAPII-S5p binding, and the PRC activities that catalyze H3K27me3 and H2Aub1. Absence of S7p and S2p at the PRC-repressed RNAPII variant may prevent cotranscriptional recruitment of RNA processing factors, leading to RNA degradation. See also Figure S3.

We find that 1,065 PRC^+^ genes are associated with RNAPII-S5p, but not S2p or 8WG16. Average ChIP-seq distributions within this cohort of repressed PRC targets identify a broad S5p promoter peak (Figure 2A), accompanied by H3K4me3 (i.e. they are bivalent; Figure S3A). S5p is detected throughout the gene body of PRC-repressed genes and decreases at the TES (Figure 2A), unlike the increase seen beyond the TES of active genes. This group of PRC targets displays no detectable S2p (Figure 2A) or H3K36me3 (Figure S3A), despite robust detection of S5p in coding regions and low-level transcripts (Figure S3B). At active genes, S5p and S7p are targeted by the same kinase (Akhtar et al., 2009; Tietjen et al., 2010). We show that the RNAPII variant at PRC-target genes is not marked by S7p (Figure S3A), raising the possibility of S5 phosphorylation by a different kinase, S7p dephosphorylation, or inaccessibility of S7 residues. Lack of S2p and S7p on PRC-repressed RNAPII may have a mechanistic role in limiting mature mRNA production by interfering with cotranscriptional recruitment of chromatin and RNA processing machinery.

The RNAPII configuration identified at PRC-repressed genes does not simply reflect uniformly lower RNAPII abundance (with a lower detection threshold for S2p than S5p). ChIP-qPCR demonstrates that S5p levels can be as high at PRC-repressed genes as at the active *β-actin* gene. However, *β-actin* is also marked by high S2p, while S2p at PRC-repressed genes is not detected above background levels (Figure 2B; Stock et al., 2007).

Strikingly, the occupancy of Ezh2 and Ring1B (Figure S3A) and their enzymatic modifications (H3K27me3 and H2Aub1; Figure 2A) are remarkably similar to that of RNAPII-S5p, being not only present at the TSS but also extending through coding regions. The presence of H3K27me3 along the coding region of PRC-target genes was recently identified in an independent study (Young et al., 2011). While single-gene analyses by ChIP-qPCR (Figure 2B) or ChIP-seq (Figure S2B) identify different extents of RNAPII elongation through coding regions of different PRC-repressed genes, they show that PRC occupancy consistently mirrors RNAPII-S5p. The similar distribution of PRC and RNAPII occupancy at this group of genes led us to ask whether RNAPII and PRCs simultaneously associate with the same chromatin at PRC-repressed TSSs and, for some genes, throughout coding regions.

### PRCs and RNAPII Physically Coassociate at Repressed Chromatin, and Positively Correlate in both Level and Distribution

To directly test whether RNAPII and PRCs simultaneously coassociate at PRC-repressed chromatin, we used sequential ChIP (re-ChIP). We first analyzed colocalization between S5p and Ring1B or Ezh2 at 18 PRC-repressed genes: (1) eight previously characterized (Stock et al., 2007) PRC^+^ S5p^+^S2p^−^ promoters (Figure S3C); (2) TSSs and TESs of six S5p^+^S2p^−^ genes, identified here as associated with S5p, three of which have S5p and Ring1B occupancy at TSSs and three at both TSSs and TESs (Figures 2B and 2C); and (3) four additional PRC targets (Figure S3C). At all 21 regions associated with both RNAPII-S5p and PRC, re-ChIP detects enrichment for RNAPII-S5p with Ring1B or Ezh2, independently of immunoprecipitation order. These results lead us to conclude that PRCs and RNAPII-S5p coassociate with chromatin, although they cannot distinguish direct from indirect interactions between the complexes. Thus, chromatin bivalency is characterized by physical coassociation of repressive (PRCs) and activating (RNAPII) enzymatic activities with chromatin, which cannot be explained by ESC heterogeneity.

The preferred association of PRC-repressed genes with RNAPII-S5p and not S2p was tested independently of ChIP assays by single-cell microscopy using immuno-cryoFISH (fluorescence in situ hybridization on ultrathin cryosections; Branco and Pombo, 2006), a high-spatial resolution, high-sensitivity imaging method (Figure 2D). PRC targets associate with S5p to extents similar to those of an active gene (*β-actin*), above the background levels observed at an inactive, PRC^−^ control (*Myf5*). They associate with S2p considerably less than *β-actin*, and to an extent similar to that of *Myf5*. These analyses confirm the association of PRC targets with RNAPII-S5p^+^S2p^−^, and show its high prevalence at the single-allele level.

To further investigate the extent of coassociation between PRCs and RNAPII, we tested whether levels of RNAPII-S5p and PRCs at individual genes are positively correlated across the ESC genome. Interdependence of RNAPII and PRCs in the PRC-repressed state is supported by strong positive correlations between S5p and H2Aub1 or H3K27me3 levels (ρ = 0.69, 0.55, respectively; Spearman's rank correlation coefficient; Figure 2E). Similar strong correlations are also seen for S5p with Ring1B or Ezh2 (ρ = 0.68, 0.68, respectively, Figure S3D). The correlations between PRCs and S5p are similar to those between the two PRC-instigated histone modifications, and between the PRC1 and PRC2 catalytic components (ρ = 0.67, 0.84, respectively; Figure S3D), supporting the significance of this interplay.

Collectively, single-gene and genome-wide analyses of the RNAPII variant identified at PRC-repressed chromatin demonstrate an unexpected molecular coassociation and synergy between the seemingly antagonistic Polycomb and RNAPII complexes. RNAPII-S5p^+^S7p^−^S2p^−^ extends throughout genes to the same extent as, and in proportion to, PRCs (Figure 2F). Absence of S2p and S7p from PRC-repressed RNAPII indicates that PRC repression involves interference with RNA processing (Figure 2F).

### Functional Derepression of PRC Targets after Ring1B Depletion

**Figure 3:**
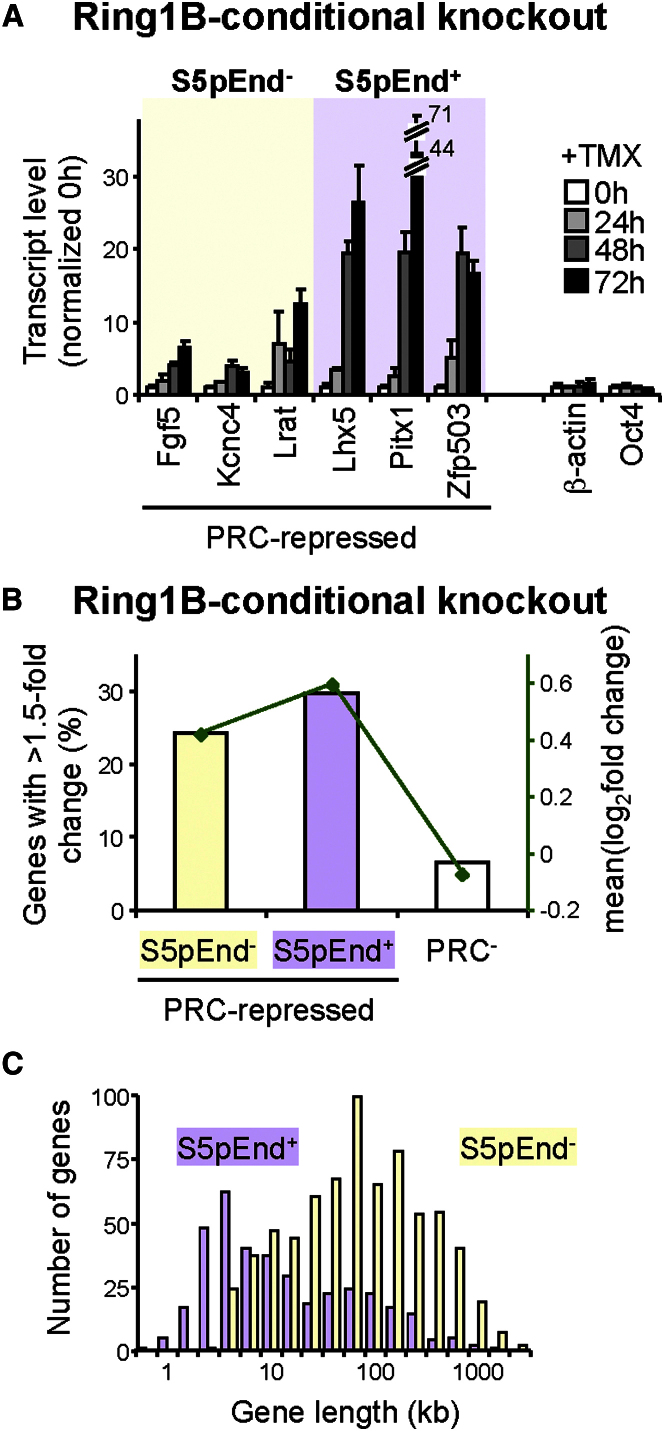
Functional PRC Repression Is Proportional to RNAPII-S5p Extension (A) PRC targets become derepressed upon Ring1B removal, with a more marked effect at genes where S5p extends up to the TES (S5pEnd^+^), than that which occurs at genes classified as S5pEnd^−^. RNA levels were measured in ES-ERT2 *Ring1A*-knockout cells after tamoxifen (TMX)-induced *Ring1B* knockout. Transcript levels were normalized to housekeeping genes, and to 0 hr. Mean and SD from three biological replicates are shown. (B) Analyses of microarray data for ES-ERT2 cells ± 48 hr TMX treatment (Endoh et al., 2008) shows that the percentage of PRC-repressed genes derepressed by >1.5-fold (bars) is significant irrespective of S5p detection at the TES (p < 10^−16^, one-tailed Fisher's exact test), although the mean fold expression change (green) is higher for genes with S5p extending to TESs (S5pEnd^+^). (C) S5pEnd^+^ PRC-repressed genes have a wide range of lengths, although the majority are shorter than those with S5p only at promoters (S5pEnd^−^; p < 2.2 × 10^−16^, one-tailed Wilcoxon rank-sum test).

To investigate the interdependence of RNAPII and PRC at PRC targets associated with RNAPII-S5p^+^S2p^−^8WG16^−^, we investigated levels of derepression upon *Ring1B* knockout. Functional repression by PRCs at this gene cohort is shown by a marked increase in transcript levels of PRC targets after inducible *Ring1B* knockout in *Ring1A* null ESCs (Figures 3A and 3B). Interestingly, single-gene studies (Figure 3A) suggested that derepression is greater for genes with S5p and PRCs extending through gene coding regions (*Lhx5*, *Pitx1*, and *Zfp503*) than for genes where both activities are more restricted to the TSS (*Fgf5*, *Kcnc4*, and *Lrat*).

Analysis of genome-wide data (Endoh et al., 2008) supports this conclusion (Figure 3B), with genes classified as positive for S5p at the TES (S5pEnd^+^) being more likely to be derepressed and show higher changes in expression levels than those for which S5p is not at the TES (S5pEnd^−^; p = 0.0026, one-tailed Wilcoxon rank-sum test). Comparison of gene length between S5pEnd^+^ and S5pEnd^−^ genes shows that S5p detection at the TES is not solely due to short gene length and promoter-proximal S5p occupancy, as 60% of S5pEnd^+^ genes are more than 5 kb long, and some are over 100 kb (Figure 3C). These results suggest that the presence of RNAPII-S5p further into coding regions of PRC-repressed genes, accompanied by proportional PRC occupancy, may favor gene activation upon PRC removal, although a contribution of shorter gene sizes cannot be excluded.

### Genome-wide Interplay between PRCs and RNAPII Variants

**Figure 4:**
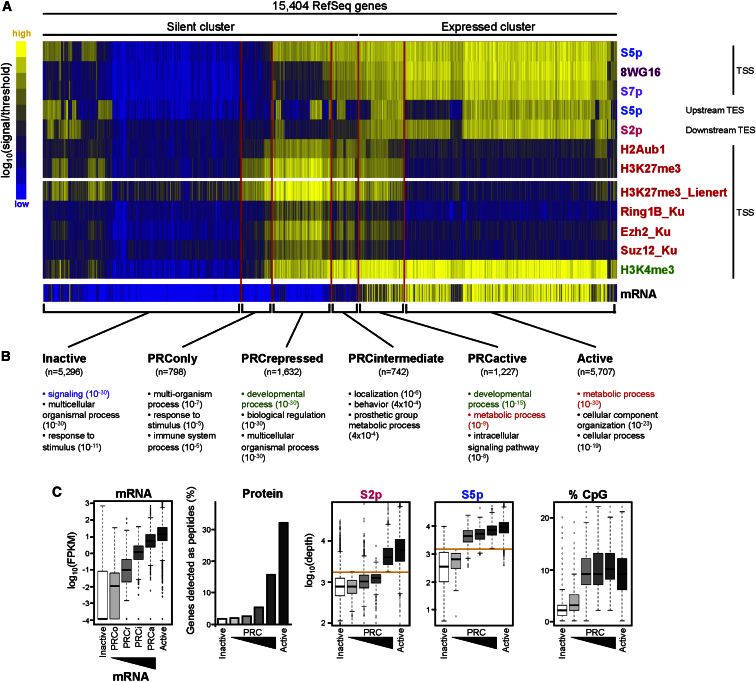
PRC Targets Associate with Different RNAPII Modifications and Expression Levels (A) Hierarchical clustering was performed after binary classification of RNAPII and PRC modifications for 15,404 nonoverlapping RefSeq genes. Marker enrichment at TSSs or TESs is normalized to the binary classification threshold. Four major PRC groups were identified: *PRConly*, *PRCrepressed*, *PRCintermediate*, and *PRCactive*. Remaining genes were classified as *Active* or *Inactive*. Levels of mRNA and additional markers are presented for comparison (lower panel), but were not used as clustering variables. (B) “Developmental process” is the most significantly enriched Gene Ontology (GO) term for *PRCr* genes, while *PRCa* terms include “developmental process” and “metabolic process” (p values in brackets, hypergeometric test). The full GO table with intergroup comparisons is shown in Table S3. (C) mRNA-seq levels are highest for *Active* genes, followed by *PRCa*, *PRCi*, *PRCr*, *PRCo*, and *Inactive*. Analysis of ESC SILAC data (Graumann et al., 2008) shows expression at the protein level only for *PRCa* and *Active* genes. S2p levels are only above background at *Active* and *PRCa*, while S5p levels are also substantial at *PRCi* and *PRCr*. Orange line, threshold. CpG content mirrors S5p enrichment. See also Figure S4.

The observation of tight interplay between PRCs and RNAPII at 1,065 silent PRC targets associated with RNAPII-S5p^+^S2p^−^8WG16^−^ led us to investigate other RNAPII variants within the whole cohort of PRC targets (5,628 genes), which includes genes with substantial mRNA expression. We used hierarchical clustering, an unbiased genome-wide approach, to identify specific combinations of PRC and RNAPII modification (Figure 4A). Genes were classified according to the presence/absence of each ChIP-seq marker in regions of interest (Table S2), in order to define discrete groups of genes with similar PRC/RNAPII states. We represent levels of H3K4me3, Ezh2, Suz12, Ring1B, and H3K27me3 for comparison (Figure 4A), the latter from a recent ChIP-seq data set (Lienert et al., 2011).

Comparing mRNA levels across the resultant gene clusters reveals clear-cut associations with expression and silencing, despite mRNA-seq data not being included as a variable in the clustering analysis (Figure 4A). This shows that presence or absence of RNAPII and PRCs can predict gene expression states in ESCs.

Within the “silent” branch of the hierarchical tree, we find three groups of PRC targets. These groups are characterized by the presence of the following: (1) H3K27me3, but little detectable H2Aub1, H3K4me3, or RNAPII (n = 798; *PRConly* or *PRCo*); (2) H3K27me3, H2Aub1, H3K4me3, and S5p, without other RNAPII modifications (n = 1,632; *PRCrepressed* or *PRCr*); and (3) H3K27me3, H2Aub1, H3K4me3, S5p, 8WG16, and S7p, but little detectable S2p or mRNA expression (n = 742; *PRCintermediate* or *PRCi*). The *PRCr* group contains all the PRC^+^ genes found to be associated with the RNAPII variant S5p^+^S2p^−^8WG16^−^ in our initial analysis (characterized in Figures 2 and 3).

Surprisingly, hierarchical clustering identifies a fourth PRC-target cluster within the “expressed” branch (n = 1,227; *PRCactive* or *PRCa*), associated with all RNAPII modifications, H3K4me3, H3K27me3, H2Aub1, and mRNA. Thus, we identify four major PRC-target gene groups: *PRConly*, *PRCrepressed*, *PRCintermediate*, and *PRCactive*. The remaining genes in the expressed and silent branches of the hierarchical tree were classified as *Active* genes (all expressed genes, excluding *PRCa*) and *Inactive* genes (silent genes minus *PRCr*, *PRCi*, and *PRCo*), respectively.

Careful inspection of ChIP-seq profiles (examples in Figure S2B) and independent validation by ChIP-qPCR (Figure S4A) confirm the different combinations of marker occupancy at single genes within each group. Single-gene qRT-PCR analyses show that transcript levels from *PRCa* genes are comparable to those of an *Active* gene, and 100- to 1,000-fold higher than *PRCr* genes (Figure S4B). Expression of PRC2-target genes has been previously described in ESCs (Nishiyama et al., 2009; Sharov et al., 2011; Young et al., 2011), and genes classified as bivalent in Mikkelsen et al. (2007) can be upregulated or downregulated upon transcription factor induction in ESCs (Nishiyama et al., 2009; Sharov et al., 2011). Here we expand on these analyses both by identifying active PRC-target genes associated with both PRC1 and PRC2, and by exploring the RNAPII state associated with them.

### Gene Ontology and KEGG Pathway Analyses Identify Roles for PRCs in Metabolic Gene Regulation

Gene Ontology (GO) analyses across the six gene groups identified by hierarchical clustering reveal enrichment for genes associated with developmental processes in *PRCr* and *PRCa*, and enrichment for signaling and response to stimuli within *Inactive* and *PRCo* (Figure 4B, see Table S3 for detailed GO analyses). This suggests that RNAPII at PRC targets may be important for gene activation during development, while PRC targets lacking RNAPII recruitment (*PRCo*) are required only in terminally differentiated cells.

Unexpectedly, *PRCa* genes are also enriched for metabolic GO terms (p < 10^−15^, hypergeometric test; Figure 4B). KEGG pathway analysis identifies *PRCa* genes associated with TGFβ-, Wnt-, and MAPK-signaling pathways, and with cancer, cell cycle, and energy metabolism (Table 1). Although deregulation of a few of the active PRC targets identified here has been reported after PRC1 knockout (van der Stoop et al., 2008), to our knowledge, direct regulation of metabolic genes by PRCs has not been shown before.

### Active PRC Targets Are Expressed at the Protein Level

To investigate the biological significance of active RNAPII modifications (S7p^+^S2p^+^) and mRNA expression at active PRC targets (*PRCa*, n = 1,227), we mined ESC proteome data (Graumann et al., 2008) to determine whether *PRCa* genes are expressed at the protein level. We positively identify peptides from 32% and 15% of *Active* and *PRCa* genes, respectively, in comparison with 2%–5% for other gene cohorts (Figure 4C). Thus, PRC occupancy is compatible with protein expression at *PRCa* genes. S2p is detected above threshold at *PRCa* and *Active* genes, while S5p is also present at *PRCr* and *PRCi*, mirroring CpG content.

Among the *PRCa* genes expressed at the protein level are transcriptional regulators (*Hdac2* and *Hmga2*), cancer-linked genes (*Klf4* and *Kit*), and genes involved in glycolysis and pyruvate metabolism (*Hk1*, *Eno2*, and *Pck2*). This suggests that PRCs modulate expression levels of active genes with important roles in ESCs, some of which are required for ESC identity [e.g., *Hmga2* (Hammond and Sharpless, 2008), *Klf4* (Takahashi and Yamanaka, 2006), and *Tbx3* (Lu et al., 2011)]. Others are involved in metabolic processes, such as glycolysis, that are differently regulated in ESCs and somatic cells (Kondoh et al., 2007).

Importantly, genes that mark early differentiation, such as *Gata4*, *Gata6*, and *Brachyury* (Singh et al., 2007), do not display detectable S2p, or other markers of productive transcription, and are categorized as *PRCr* (Figure S5A). This supports the conclusion that the *PRCa* cohort of genes is related to the pluripotent state and is not due to differentiation in our ESC cultures. Single-cell immunofluorescence analyses show Oct4 and Nanog detection across the population of ESCs, albeit at variable levels (Figure 1A, Figure S5B).

### Mechanisms of PRC Control at Active PRC Targets

**Figure 5:**
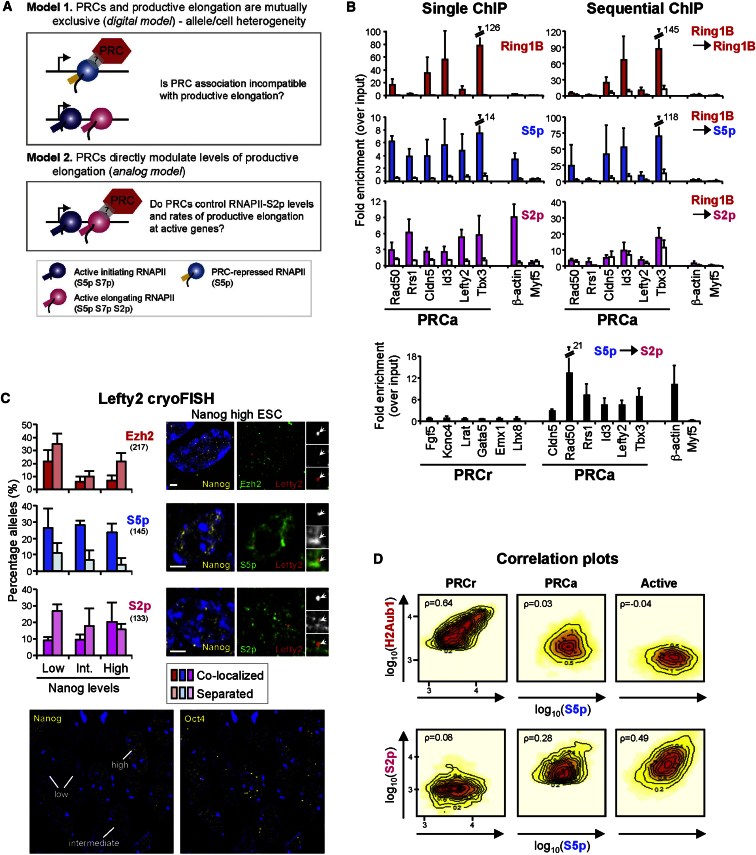
PRCs and Elongating RNAPII-S2p Are Mutually Exclusive at Active PRC-Target Genes (A) Two alternative models of PRC regulation at *PRCa* genes. (B) Ring1B, S5p, and S2p occupancy and coassociation at *Active* (*β-actin*), *Inactive* (*Myf5*), and *PRCa* genes were analyzed by ChIP or re-ChIP and qPCR, as described in Figure 2. Mean and SD from two to five biological replicates are shown. ChIP-qPCR confirms binding of Ring1B, S5p, and S2p to *PRCa* genes, but re-ChIP shows PRC1 coassociation with S5p, but not with S2p, above background levels (white bars). Re-ChIP demonstrates simultaneous presence of S2p and S5p at *PRCa* and *Active*, but not *PRCr*, genes. (C) Colocalization of *PRCa* gene *Lefty2* (red, arrows) with sites containing S5p, S2p, or Ezh2 (green) was measured by immuno-cryoFISH in ESC nuclei with different levels of Nanog (yellow; classified as high, low, or intermediate). Locus association with each marker was scored as colocalized (≥1 pixel overlap) or separate. *Lefty2* associates with S5p at similar frequency regardless of Nanog status, but association with S2p is highest and Ezh2 is lowest in Nanog^high^ cells. Bar: 2 μm. The number of loci analyzed is indicated in brackets. Note that all cells were positive for Oct4 despite variable levels of Nanog (lower panel). (D) Correlation plots for enrichment levels in 2 kb windows for *PRCr*, *PRCa*, and *Active* clusters. Positive correlations are stronger between S5p and H2Aub1 within *PRCr* and between S5p and S2p at *PRCa* genes (ρ, Spearman's rank correlation coefficient). See also Figure S5.

To investigate the mechanism of PRC function at *PRCa* genes, we asked whether coexistence of PRC repression and RNAPII productive transcription could be due to separate chromatin states in different alleles across the heterogeneous ESC population (Figure 5A, *Model 1*), or whether PRCs directly associate with active RNAPII-S2p complexes (Figure 5A, *Model 2*). Coassociation between S5p, S2p, and PRCs would be different in the two models. In *Model 1*, RNAPII-S5p and PRC are present at the PRC-bound allele, but PRCs are not at the active allele; in this case, PRCs would re-ChIP with S5p, but not S2p. In *Model 2*, RNAPII-S5p, S2p, and PRCs are simultaneously bound to the same chromatin and PRCs would re-ChIP with both S5p and S2p.

We performed sequential ChIP of Ring1B with RNAPII-S5p or S2p to test these models (Figure 5B). Notably, Ring1B coassociates with S5p-bound chromatin but does not colocalize with S2p above background levels. This suggests that PRCs coassociate with RNAPII-S5p at *PRCa* genes, but antagonize phosphorylation of S2. Therefore the two states, PRC-repressed and active, exist separately within a cell (binding to different alleles) or cell population.

Lack of coassociation between PRCs and S2p thus supports an “on-off” (digital) switch mechanism of PRC regulation, where PRC impedes establishment of active RNAPII at PRC-bound chromatin (Figure 5A, *Model 1*). Ring1B coassociation with RNAPII-S5p at *PRCa* (Figure 5B) and *PRCi* (Figure S3C) genes shows that PRCs colocalize with RNAPII-S5p at all RNAPII-associated PRC targets. Re-ChIP experiments of S5p with S2p confirm the presence of S5p with S2p at actively transcribed genes within both *PRCa* and *Active* groups, but not at *PRCr genes* (Figure 5B).

Next, we explored whether the independent association of *PRCa* genes with PRC and S5p (in the PRC-repressed state), or with S2p and S5p (in the active state), could be related to natural fluctuations in transcription factor levels across the heterogeneous ESC population. We performed single-cell cryoFISH colocalization of the *PRCa* gene *Lefty2* with RNAPII-S5p, RNAPII-S2p, and Ezh2 (PRC2) in ESCs costained with Nanog antibodies (Figure 5C). Interestingly, these experiments show similar association of the *Lefty2* locus with RNAPII-S5p independent of Nanog levels, but a significant association with RNAPII-S2p in Nanog^high^ cells and with Ezh2 in Nanog^low^ cells (p = 0.2 [S5p], 0.006 [S2p], and 0.04 [Ezh2]; χ^2^ test). These studies suggest that pluripotency transcription factors that fluctuate within the ESC population, such as Nanog, may influence the switch between PRC-repressed and active states of *PRCa* genes.

To complement the analyses of single genes by sequential ChIP and cryoFISH, we investigated genome-wide correlations between S5p and H2Aub1 or S2p. Consistent with *Model 1*, the correlation between S5p and PRCs in the *PRCa* cohort is lower than in the *PRCr* state (Figure 5D), suggesting that the association between S5p and PRCs is diluted by the presence of some S5p complexes associated only with S2p. Furthermore, S2p levels correlate more extensively with S5p at *PRCa* than at *PRCr* (Figure 5D), in agreement with the presence of a population of *PRCa* genes with S5p and S2p.

These studies support a switch model of active PRC-target genes (*Model 1*, Figure 5A), where *PRCa* genes are not simultaneously repressed by PRCs and expressed. At active *PRCa* genes, RNAPII-S5p exists in two independent states: (1) in association with PRC (in the absence of other RNAPII active marks) and (2) in the presence of S2p (and absence of PRCs). These data suggest that *PRCa* genes, which in population-based ChIP analyses appear to be expressed and bound by PRCs, are not simultaneously expressed and under PRC repression at the single-gene level. This may be due to allelic exclusion or ESC heterogeneity. Further studies will be necessary to understand the means by which pluripotency transcription factors influence fluctuations between epigenetic states at this important cohort of PRC targets.

### Expression Levels of Active PRC Targets Are Regulated by PRCs

**Figure 6:**
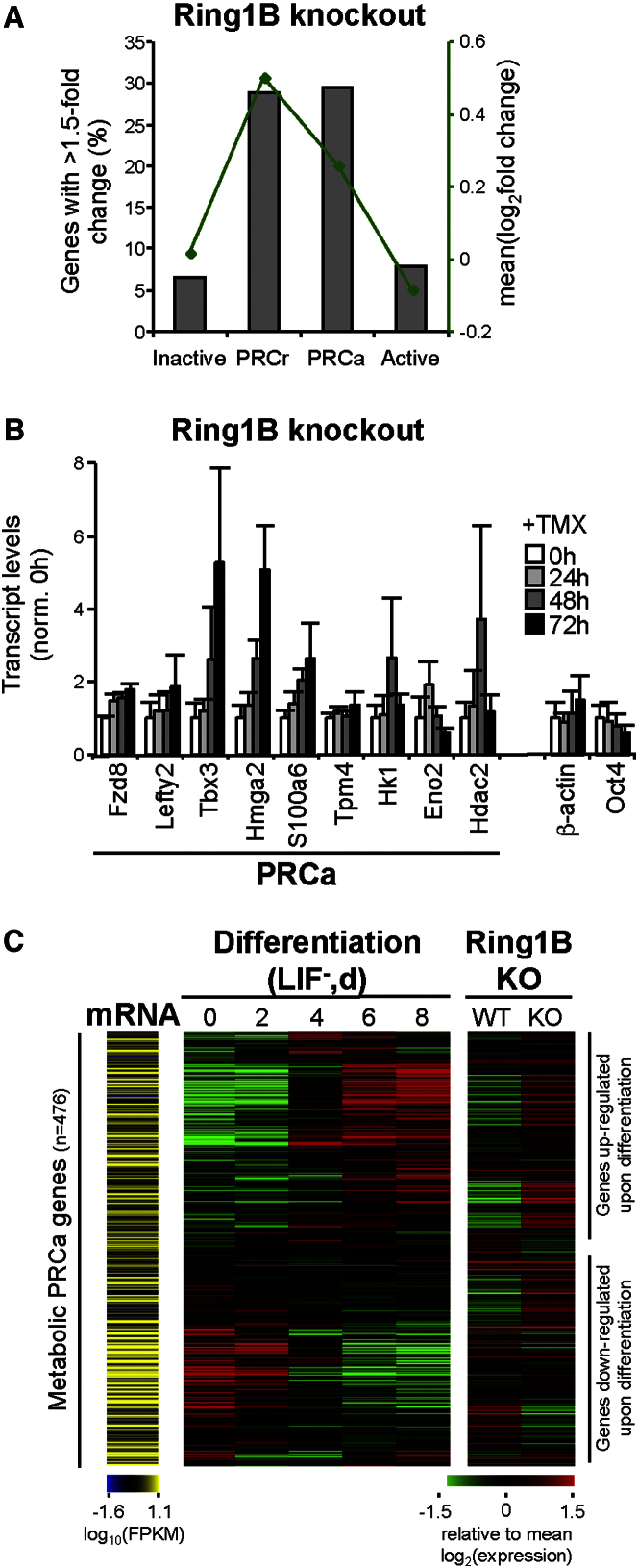
PRC1 Functionally Represses Active Developmental Genes and Metabolic Genes in ESCs (A) Analysis of microarray data for ES-ERT2 cells ±48 hr TMX treatment (Endoh et al., 2008) shows that *PRCa* genes undergo derepression after Ring1B removal. Percentage of genes showing >1.5-fold increase is statistically significant for *PRCa* and *PRCr* (p < 10^−53^, one-tailed Fisher's exact test). Mean fold change (green) is lower for *PRCa* than *PRCr*. (B) Single-gene qRT-PCR analyses, as described in Figure 3, show functional derepression of *PRCa* genes upon Ring1B removal in ES-ERT2 cells. Mean and SD from three biological replicates are shown. (C) Analyses of microarray expression data for ESC differentiation after LIF withdrawal (Shen et al., 2009) and *Ring1B* knockout in ES-ERT2 cells (Endoh et al., 2008) show that metabolic *PRCa* genes can become upregulated or downregulated upon differentiation. 476 *PRCa* genes with “metabolic process” GO are represented (GO:0008152). Red/green colors represent expression changes relative to the mean across the gene group represented. *PRCa* genes are expressed in ESCs (differentiation d0), as illustrated by mRNA-seq data (expression normalized for total RefSeq genes). Genes are ordered according to hierarchical clustering of microarray data for differentiation. See also Figure S6.

To investigate whether *PRCa* genes are functionally repressed by the presence of PRC, we repeated our meta-analyses of microarray expression data after conditional *Ring1B* knockout (Endoh et al., 2008) for this group of genes (Figure 6, Figure S6). We compared the changes in expression that occur at active PRC targets (*PRCa*) with the changes occurring in the well-characterized cohort of silent developmental PRC-target genes (identified here as the *PRCr* group).

Functional PRC-mediated repression of active PRC targets was confirmed, as the same proportion (∼30%) of *PRCa* and *PRCr* genes show substantial derepression after *Ring1B* knockout (p < 10^−54^, one-tailed Fisher's exact test; Figure 6A). The mean expression change at *PRCa* genes after *Ring1B* knockout is lower than at *PRCr* genes (p = 0.022, one-tailed Wilcoxon rank-sum test), likely due to the fact that *PRCa* genes are already expressed in ESCs.

Importantly, derepression of *PRCa* genes after conditional *Ring1B* knockout, together with the detection of PRC marks, adds further evidence to their classification as bona fide PRC targets. PRC1 repression of active PRC targets was further validated by qRT-PCR of single-gene transcripts over a *Ring1B* knockout time course (Figure 6B); these include important genes for ESC biology (*Hmga2*, *Tbx3*, and *Hdac2*), development (*Fzd8* and *Lefty2*), and metabolism (*Hk1* and *Eno2*). In support of our findings, three genes (*Tbx3*, *Klf4*, and *Foxd3*) here classified as *PRCa* were also recently described as PRC2 targets (Walker et al., 2010).

To probe the role of active PRC-target genes in pluripotency or during differentiation, we analyzed microarray expression data during ESC differentiation following LIF withdrawal (Shen et al., 2009; Figure 6C, Figure S6). Downregulation of many metabolic *PRCa* genes during differentiation (Figure 6C), including genes involved in glycolysis and pyruvate metabolism (*Ldha*, *Gpd1l*, and *Pck2*; Table 1), suggests that PRC1 controls the expression of genes specifically associated with pluripotency. Conversely, other metabolic *PRCa* genes become upregulated during differentiation (Figure 6C), suggesting roles during lineage specification.

Aligning *Ring1B* knockout expression data to these differentiation analyses demonstrates that metabolic *PRCa* genes that become derepressed upon Ring1 depletion can be upregulated or downregulated upon differentiation (Figure 6C); the pattern is similar across all *PRCa* genes (Figure S6). Regulation of PRC-repressed metabolic genes during ESC differentiation expands our understanding of Polycomb function in pluripotency to include the modulation of ESC metabolism, in addition to repression of developmental genes.

## Discussion

In summary, we present advanced analyses of Polycomb repression and RNAPII states in ESCs, which combine molecular, cellular, and genomic techniques on single-gene and genome-wide scales. We identify cohorts of PRC-associated genes with distinct RNAPII and expression states.

At silent developmental PRC targets, PRCs are tightly interlinked with RNAPII-S5p complexes at promoters and throughout coding regions, which produce transcripts that do not mature into mRNA, and from which protein is not produced. We demonstrate that chromatin bivalency is a phenomenon of this coassociation and synergy between PRCs and RNAPII.

At active PRC targets, PRCs are also tightly interlinked with unproductive RNAPII-S5p complexes. We demonstrate that active PRC targets can switch between PRC-repressed and active states within the ESC population. Thus, genes characterized by PRCs and expression (mRNA, protein, elongating RNAPII, and H3K36me3) are not simultaneously bound by PRCs and expressed. This may be due to allelic differences or cellular heterogeneity. The active cohort of PRC targets is enriched for genes with ontologies related to development or metabolism, and involved in metabolic processes and signaling pathways that are important for ESC biology.

PRC repression is therefore associated with a single RNAPII state (S5p^+^S7p^−^S2p^−^) across all CpG-rich genes. Fluctuation from the PRC-repressed to the canonical active state (S5p^+^S7p^+^S2p^+^) occurs to variable extents across different PRC targets, resulting in differing expression levels.

Direct modulation of metabolic and developmental genes by PRCs is likely crucial in specifying effective programs of gene expression and metabolic control that are important for ESC pluripotency and lineage specification.

## Experimental Procedures

A detailed description of materials and methods is given in Supplemental Information.

### Cell Culture

Mouse ES-OS25, ES-ERT2, and XEN cells were grown as previously described (Stock et al., 2007). For *Ring1B* conditional deletion, ES-ERT2 cells were cultured in 800 nM 4-hydroxy-tamoxifen.

### Chromatin Immunoprecipitation

ChIP assays were performed essentially as described previously (Stock et al., 2007). Sequential ChIP was performed as standard fixed ChIP, with elutions after the first immunoprecipitation in small volumes (total 80 μl) to allow dilution of SDS back to 0.1% prior to the second immunoprecipitation. Enrichment was calculated relative to the original input using the same amount of DNA in the PCRs.

### RNA Purification and qRT-PCR Analysis

Total RNA was isolated using TRIzol (Invitrogen) extraction following the manufacturer's instructions and immediately treated with TURBO DNase I (Ambion). Treated RNA was reverse transcribed using random primers.

### Illumina High-Throughput Library Preparation and Sequencing

ChIP-seq libraries were prepared according to Illumina protocols (Part #11257047 Rev A), with modifications: samples were PCR amplified prior to size selection. mRNA-seq library was prepared from total RNA after TRIzol extraction, according to Illumina's instructions (#1004898 Rev A) with some modifications. After polyA selection, ribosomal RNA was depleted using the Ribominus kit (Invitrogen). Libraries were quantified by Qubit (Invitrogen) and qPCR, and library size was assessed by Bioanalyzer (Agilent). Libraries were sequenced using an Illumina Genome Analyzer II.

### ChIP-seq and RNA-seq Analysis

Table S1 lists new and publicly available ChIP-seq data sets analyzed. Sequenced reads were aligned to the UCSC mouse mm9 genome. mRNA-seq reads were aligned to the mm9 genome and UCSC annotated transcripts (UCSC Known Gene annotations) using Tophat v.1.0.13 (Trapnell et al., 2009) and Cufflinks v.0.8.2 (Trapnell et al., 2010), to detect reads crossing exon-exon junctions and allow calculation of FPKM levels.

To investigate RNAPII modifications or PRC genome-wide, we classified each gene as positive or negative for each marker at TSSs and/or TESs (Table S2).

For hierarchical clustering, the input matrix was composed of 15,404 nonoverlapping RefSeq genes and 7 binary variables: S5p (±1 kb TSS), 8WG16 (TSS), S7p (TSS), S5p (2 kb downstream TES), S2p (2 kb upstream TES), H3K27me3 (TSS), and H2Aub1 (TSS). All pairwise dissimilarities in the data matrix were computed using the Gower coefficient; hierarchical clustering was calculated using average linkage and the function *hclust* in R.

Log_10_ transformation was applied before plotting in R heatmaps, boxplots, and correlations; a pseudocount of 1 or 0.0001 was added prior to the logarithm transformation for ChIP-seq or mRNA-seq FPKM levels, respectively, unless otherwise stated.

### GO and KEGG Pathway Analyses

Analysis of GO functional enrichment was performed using the Fisher's exact test implemented in the topGO Bioconductor package (Alexa et al., 2006). The annotation of GO terms to Entrez gene IDs was provided from the Bioconductor package org.Mm.eg.db (version 2.4.1; Gentleman et al., 2004).

Annotation of KEGG pathways and their associated genes were retrieved from ftp://ftp.genome.jp/pub/kegg/ (Kanehisa and Goto, 2000). Enrichment of KEGG pathways was assessed by hypergeometric testing in R Stats package and false discovery rates were calculated using R Multtest.

### Immuno-cryoFISH

CryoFISH was performed as previously described (Branco and Pombo, 2006; Ferrai et al., 2010).

## Figures and Tables

**Table 1:** KEGG Pathways Significantly Enriched in Active PRC-Target Genes, or the *PRCa* Cluster

KEGG Pathway (p value)	Gene Symbols (PRCa Gene Members)
Pathways in cancer (2 × 10^−6^)	*Stat1*, *Gli2*, *Ralb*, *Rassf5*, *Hdac2*, *Bcr*, *Fgf18*, *Tcf7*, *Stat5b*, *Lamb1*, *Egln3*, *Traf3*, *Akt1*, *Fgf17*, *Myc*, *Pdgfb*, *Cdkn1a*, *Vegfa*, *Lama1*, *Epas1*, *Fzd8*, *Vegfb*, *Rxra*, *Plcg1*, *Nfkb1*, *Tgfbr1*, *Cdkn2b*, *Cdk6*, *Kit*, *Fzd10*, *Pdgfa*, *Smo*, *Tcf7l1*, *Tgfb1*, *Igf1r, Fgf3*, *Ccnd1*, *Fgfr1*, *Pik3r2*, *Mmp2*, *Plcg2*, *Smad3*
TGF-beta signaling pathway (4 × 10^−6^)	*Bmpr2*, *Inhbb*, *Lefty2*, *Lefty1*, *Id2*, *Id4*, *Myc*, *Id1*, *Bmp7*, *Tgfbr1*, *Cdkn2b*, *Bmp8a*, *Id3*, *Tgfb1*, *Tfdp1*, *Smad3*, *Smad6*
MAPK signaling pathway (1 × 10^−5^)	*Mapkapk2*, *Gadd45b*, *Fgf18*, *Dusp14*, *Cacnb1*, *Dusp3*, *Map3k14*, *Map3k9*, *Akt1*, *Gadd45 g*, *Flnb*, *Nfatc4*, *Fgf17*, *Myc*, *Pdgfb*, *Mapk12*, *Mapk11*, *Mapk8ip2*, *Mapk4*, *Rasgrp2*, *Rps6ka4*, *Dusp5*, *B230120H23Rik*, *Cdc25b*, *Pla2g12a*, *Nfkb1*, *Mos*, *Tgfbr1*, *Pdgfa*, *Tgfb1*, *Fgf3*, *Fgfr1*, *Dusp4*, *Rasa2*, *Mras*, *Cacna2d2*, *Dusp9*
Cell cycle (1 × 10^−3^)	*Hdac2*, *Gadd45b*, *Gadd45 g*, *Cdc14b*, *Myc*, *Smc1b*, *Cdkn1a*, *Cdc25b*, *Cdkn2b*, *Sfn*, *Cdk6*, *Tgfb1*, *Ccnd1*, *Tfdp1*, *Smad3*
Wnt signaling pathway (1 × 10^−3^)	*Vangl2*, *Camk2b*, *Tcf7*, *Ppp2r5c*, *Sfrp4*, *Nkd2*, *Nfatc4*, *Myc*, *Fzd8*, *Lrp5*, *Fosl1*, *Frat1*, *Sfrp2*, *Vangl1*, *Fzd10*, *Tcf7l1*, *Ccnd1*, *Sfrp1*, *Smad3*
p53 signaling pathway (3 × 10^−3^)	*Steap3*, *Gadd45b*, *Igfbp3*, *Gadd45 g*, *Cdkn1a*, *Pmaip1*, *Zmat3*, *Sfn*, *Cdk6*, *Ccnd1*
Inositol phosphate metabolism (6 × 10^−3^)	*Synj2*, *Pip5k1b*, *Pip4k2a*, *Pip5kl1*, *Plcg1*, *Pik3c2a*, *Plcg2*
Pyruvate metabolism (0.01)	*Pck2*, *Acss1*, *Akr1b3*, *Ldhb*, *Ldha*, *Dlat*
Notch signaling pathway (0.01)	*Hdac2*, *Jag1*, *Hes5*, *Lfng*, *Dll3*, *Aph1c*
ErbB signaling pathway (0.02)	*Camk2b*, *Stat5b*, *Akt1*, *Myc*, *Cdkn1a*, *Hbegf*, *Plcg1*, *Pik3r2*, *Gab1*, *Plcg2*
Citrate cycle (TCA cycle) (0.04)	*Ogdh*, *Pck2*, *Dlat*
Glycolysis/ gluconeogenesis (0.04)	*Hk1*, *Pck2*, *Acss1*, *Eno2*, *Ldhb*, *Ldha*, *Dlat*

Enrichment of KEGG pathways by group members was assessed by hypergeometric testing. See also Table S3.

## Accession Numbers

ChIP-seq and mRNA-seq data have been submitted to the GEO repository under accession number GSE34520.

## Supplemental Information

Document S1. Figures S1–S6, Tables S1 and S3, and Supplemental Experimental Procedures

Table S2. Genome-wide Chromatin and RNAPII States in Mouse ESCs Mouse UCSC Known Genes (mm9) are listed, with gene classifications occupancy of RNAPII modifications, PRC components, histone modifications, and mRNA expression levels. Classifications are made into six gene clusters according to hierarchical clustering.
