# Supplementary material for: Polycomb Associates Genome-wide with a Specific RNA Polymerase II Variant, and Regulates Metabolic Genes in ESCs
Source: Cell Stem Cell. 2012 Feb 3;10(2):157–70. doi: 10.1016/j.stem.2011.12.017 (PMC3682187; doi:10.1016/j.stem.2011.12.017)
Supplement: Document S1. Figures S1–S6, Tables S1 and S3, and Supplemental Experimental Procedures [file mmc1.pdf]

Cell Stem Cell, volume *10*

Supplementary Information

Polycomb Associates Genome-wide with a Specific RNA Polymerase II Variant, and Regulates Metabolic Genes in ESCs

Emily Brookes, Inês de Santiago, Daniel Hebenstreit, Kelly J. Morris, Tom Carroll, Sheila Q. Xie, Julie K. Stock, Martin Heidemann, Dirk Eick, Naohito Nozaki, Hiroshi Kimura, Jiannis Ragoussis, Sarah A. Teichmann, Ana Pombo

Inventory of Supplementary Information

1. Supplementary Figures S1-S6

Supplementary Figure S1. Mapping PRCs and histone modifications to investigate the extent of chromatin bivalency – related to Figure 1.

Supplementary Figure S2. ChIP-seq profiles across single genes reveal different combinations of RNAPII and histone modifications at active, inactive and PRC-target genes – related to Figure 1.

Supplementary Figure S3. Interplay between Polycomb and RNAPII-S5p at PRC-repressed S5p-bound genes – related to Figure 2.

Supplementary Figure S4. Single gene analyses confirm the RNAPII modifications and expression status of genes from distinct PRC groups – related to Figure 4.

Supplementary Figure S5. Active PRC-target genes do not represent ESC differentiation – related to Figure 5.

Supplementary Figure S6. *PRCa* and *PRCr* genes show de-repression upon *Ring1B* knockout but can be up- or down-regulated upon differentiation – related to Figure 6.

2. Supplementary Tables S1 and S3

Supplementary Table S1. ChIP-seq datasets used in this study.

Supplementary Table S3. Enrichment in Gene Ontology (GO) terms at Active PRC-target and Inactive clusters – related to Table 1.

3. Supplementary Text

Supplementary Methods

Supplementary References

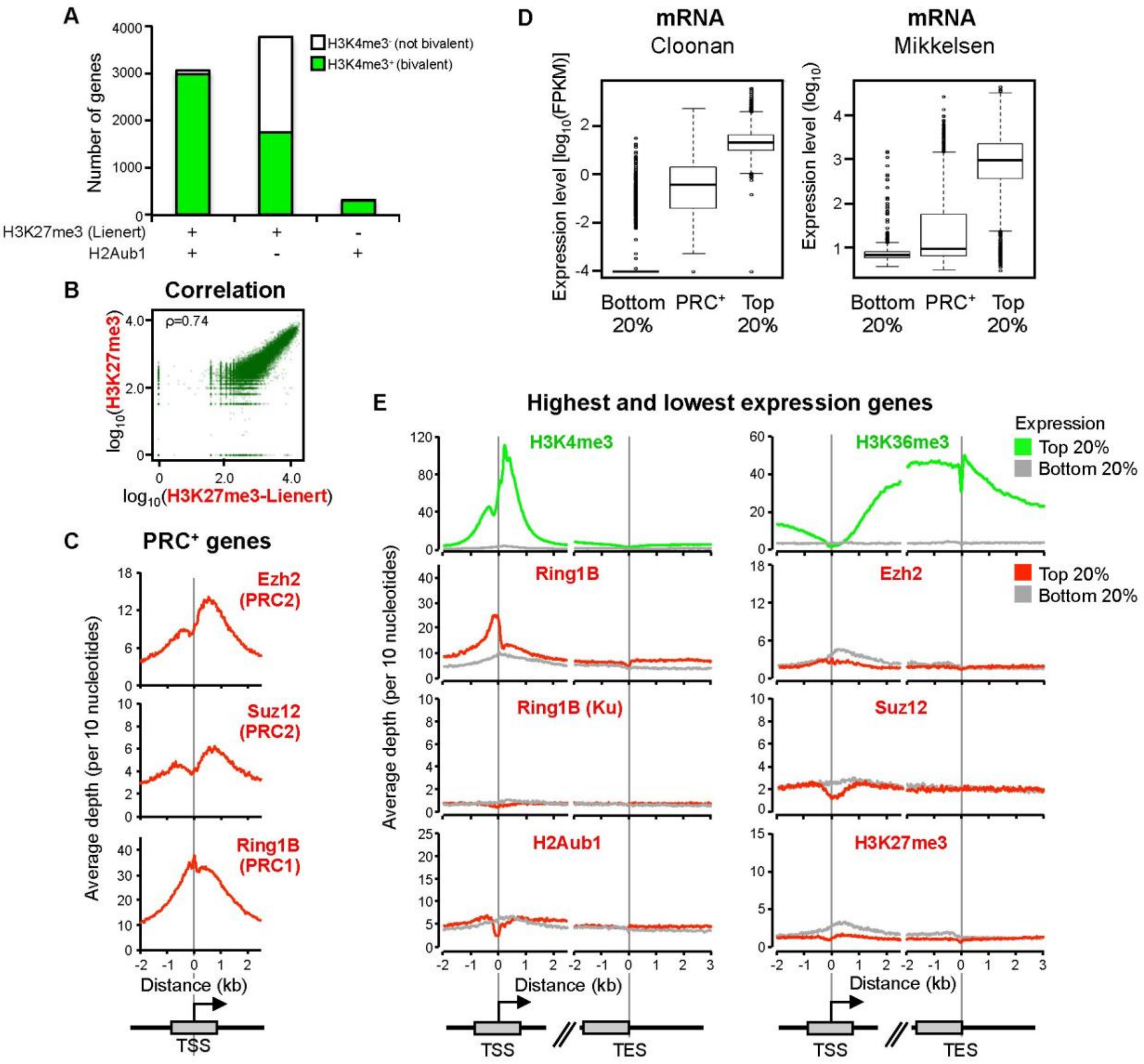
A
Number of genes
H3K4me3- (not bivalent)
H3K4me3+ (bivalent)
H3K27me3 (Lienert)
H2Aub1
B
Correlation
ρ=0.74
log10(H3K27me3)
log10(H3K27me3-Lienert)
C
PRC+ genes
Ezh2 (PRC2)
Suz12 (PRC2)
Ring1B (PRC1)
Average depth (per 10 nucleotides)
Distance (kb)
TSS
D
mRNA Cloonan
mRNA Mikkelsen
Expression level [log10(FPKM)]
Expression level (log10)
Bottom 20%
PRC+
Top 20%
E
Highest and lowest expression genes
Expression
Top 20%
Bottom 20%
H3K4me3
H3K36me3
Ring1B
Ezh2
Ring1B (Ku)
Suz12
H2Aub1
H3K27me3
Average depth (per 10 nucleotides)
Distance (kb)
TSS
TES

Supplementary Figure S1. Mapping PRCs and histone modifications to investigate the extent of chromatin bivalency – related to Figure 1.

A, Classifying genes using H2Aub1, H3K27me3_Lienert, and H3K4me3, reveals H3K4me3 to be more associated with H2Aub1 than H3K27me3, as seen using H3K27me3_Mikkelsen data (Fig. 1B).

B, Good correlation between H3K27me3 enrichment in 2kb TSS windows using ChIP-seq data from Mikkelsen et al. (2007) or Lienert et al. (2011).

C, Occupancy of PRC subunits was assessed at PRC^+ ($H3K27me3^+$ and/or $H2Aub1^+$) genes. PRC1 subunit, Ring1B, and PRC2 subunits, Ezh2 and Suz12, occupy promoter regions with a similar distribution to their histone modifications (H2Aub1 and H3K27me3, respectively; Fig. 1C).

D, mRNA expression levels were determined for the group of 20% highest expressed, 20% lowest expressed and PRC^+ genes using published mRNA-seq (Cloonan et al., 2008) and microarray (Mikkelsen et al., 2007) data. PRC^+ genes show a wide range of expression levels.

E, Distribution of PRC subunits and active or repressive histone modifications was assessed for the 20% highest (colors) and lowest (grey) expressed genes. Active genes are marked by H3K4me3 at promoters and H3K36me3 in coding regions, but not by PRC proteins or histone modifications. Inactive genes do not display PRCs, H3K4me3 or H3K36me3. Ring1B displays an unexpected, non-specific peak at the most active genes; this Ring1B data was explored further due to the low sequencing depth of published Ring1B data. PRC1 target classification was based on H2Aub1 to minimize misclassification.

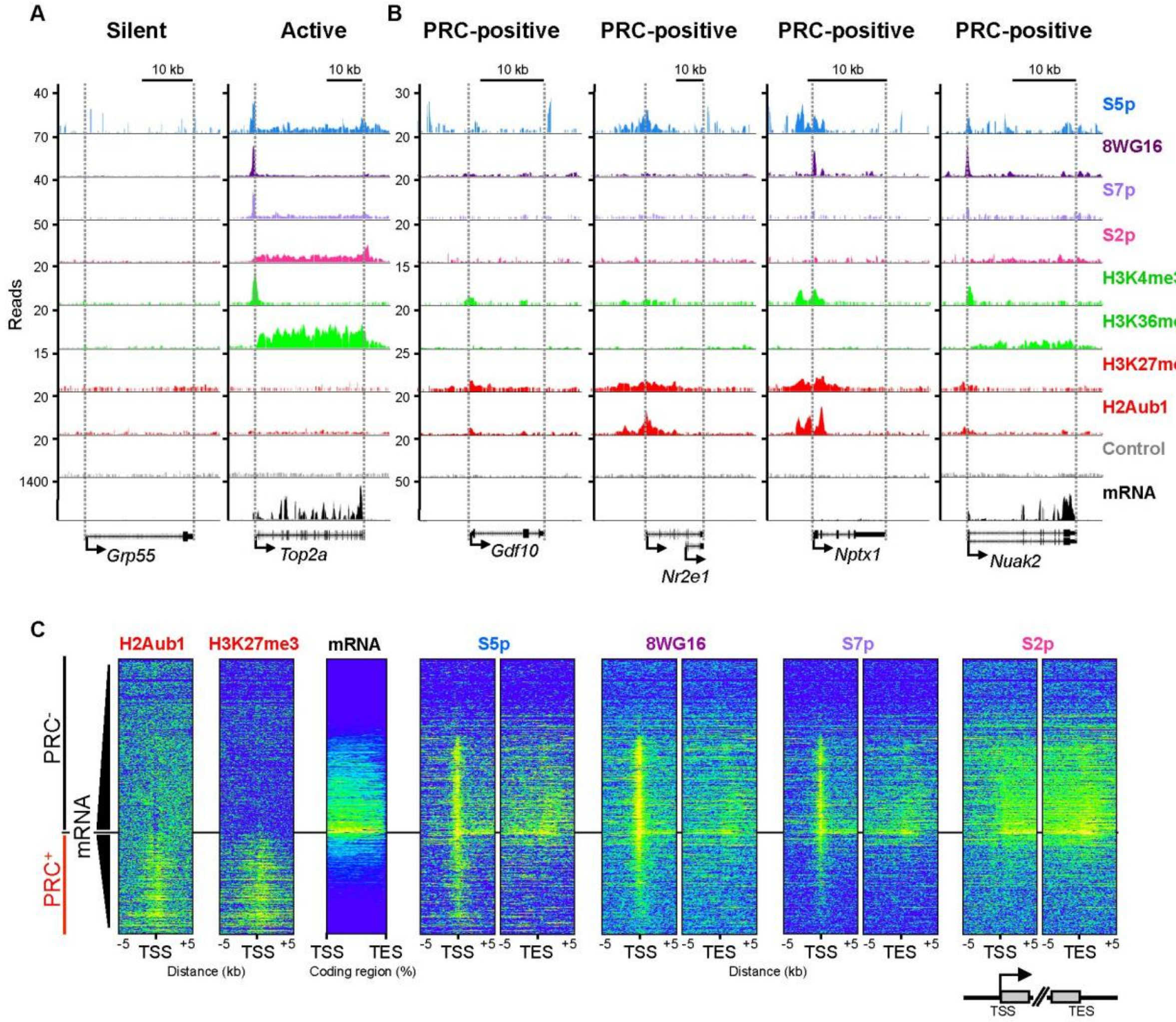
A
Silent
Active
B
PRC-positive
PRC-positive
PRC-positive
PRC-positive
10 kb
Reads
S5p
8WG16
S7p
S2p
H3K4me3
H3K36me3
H3K27me3
H2Aub1
Control
mRNA
Grp55
Top2a
Gdf10
Nr2e1
Nptx1
Nuak2
C
H2Aub1
H3K27me3
mRNA
S5p
8WG16
S7p
S2p
PRC-
PRC+
TSS
TES
Distance (kb)
Coding region (%)

Supplementary Figure S2. ChIP-seq profiles across single genes reveal different combinations of RNAPII and histone modifications at active, inactive and PRC-target genes – related to Figure 1.

A, At active genes, S5p, S7p, 8WG16 and H3K4me3 show prominent promoter peaks, while S2p and H3K36me3 increase through coding regions. S2p peaks beyond the TES.

B, At PRC-target genes, S5p, H3K27me3 and H2Aub1 show similar enrichment domains. At some PRC-positive genes, low levels of mRNA and S2p are detected. The PRC-positive genes displayed were later classified as *PRConly* (*Gdf10*), *PRCrepressed* (*Nr2e1*), *PRCintermediate* (*Nptx1*), and *PRCactive* (*Nuak2*).

C, ChIP-seq density heatmaps for 15,404 non-overlapping RefSeq genes classified as PRC-negative (PRC^-, n=9776) or PRC-positive (PRC^+, n=5628) and ordered by mRNA-seq levels. PRC^+ genes include both silent and active genes, and associate with S5p irrespective of activity.

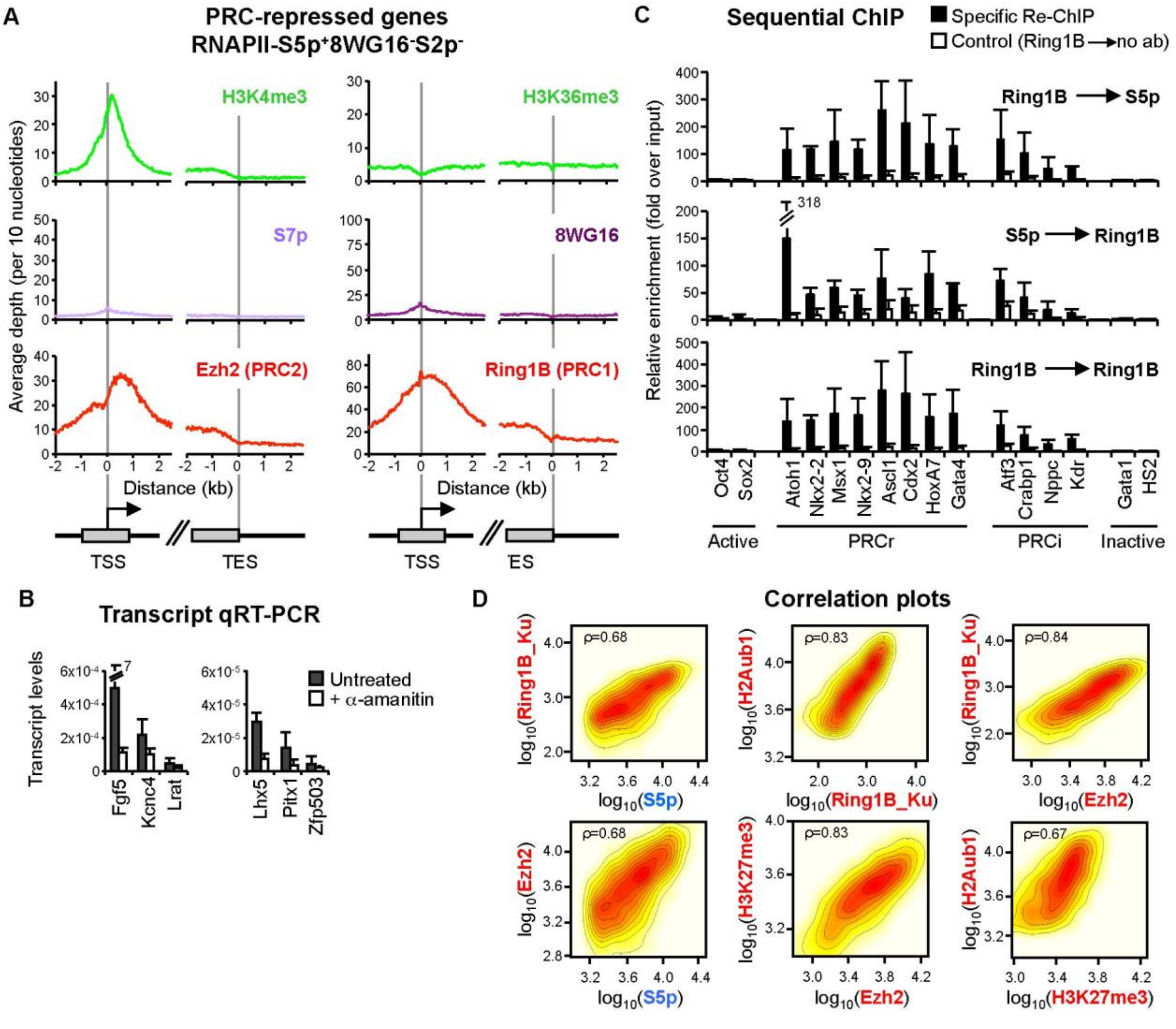
A
PRC-repressed genes
RNAPII-S5p+8WG16-S2p-
H3K4me3
H3K36me3
S7p
8WG16
Ezh2 (PRC2)
Ring1B (PRC1)
Average depth (per 10 nucleotides)
Distance (kb)
TSS
TES
B
Transcript qRT-PCR
Transcript levels
Untreated
+ α-amanitin
Fgf5
Kcnc4
Lrat
Lhx5
Pitx1
Zfp503
C
Sequential ChIP
Specific Re-ChIP
Control (Ring1B → no ab)
Ring1B → S5p
S5p → Ring1B
Ring1B → Ring1B
Relative enrichment (fold over input)
Oct4
Sox2
Atoh1
Nkx2-2
Msx1
Nkx2-9
Ascl1
Cdx2
HoxA7
Gata4
Atf3
Crabp1
Nppc
Kdr
Gata1
HS2
Active
PRCr
PRCi
Inactive
D
Correlation plots
ρ=0.68
ρ=0.83
ρ=0.84
ρ=0.68
ρ=0.83
ρ=0.67
log10(Ring1B_Ku)
log10(S5p)
log10(H2Aub1)
log10(Ring1B_Ku)
log10(Ezh2)
log10(H3K27me3)

Supplementary Figure S3. Interplay between Polycomb and RNAPII-S5p at PRC-repressed S5p-bound genes – related to Figure 2.

A, Distribution of markers was assessed at 1065 PRC-repressed genes ($H3K27me3^{+}H2Aub1^{+}S5p^{+}$ $S2p^{-}8WG16^{-}$). H3K4me3 is detected at promoters and into coding regions (consistent with S5p; Fig. 2A). H3K36me3 is not detected (consistent with S2p; Fig. 2A). S7p and 8WG16 are detected only at low levels. Ring1B and Ezh2 profiles are very similar to those of H2Aub1 and H3K27me3 (Fig. 2A), respectively.

B, Total RNA levels measured using primers located at gene 5'-ends, and normalized to housekeeping genes. Open bars, alpha-amanitin treatment (7h, 75 µg/ml) to inhibit transcription. Transcripts are detected at PRC-repressed genes, shown by sensitivity to alpha-amanitin. Mean and SD from 3 biological replicates.

C, Sequential ChIP of Ring1B followed by RNAPII-S5p (or *vice versa*) demonstrates co-localization of these two proteins at promoter regions of PRC targets (black bars; 12/12 genes). Background levels (mean enrichment after first ChIP with Ring1B and re-ChIP with no antibody) are presented (white bars) next to each data point. Mean and SD from 3 biological replicates.

D, Positive correlation between S5p and Ring1B or Ezh2 levels within 2kb TSS windows of PRC-repressed genes (ρ, Spearman's rank correlation coefficient), supports positive correlation between S5p and H2Aub1 or H3K27me3 (Fig. 2E). The extent of PRC correlation with S5p is similar to that between PRC components and histone modifications, supporting its biological relevance.

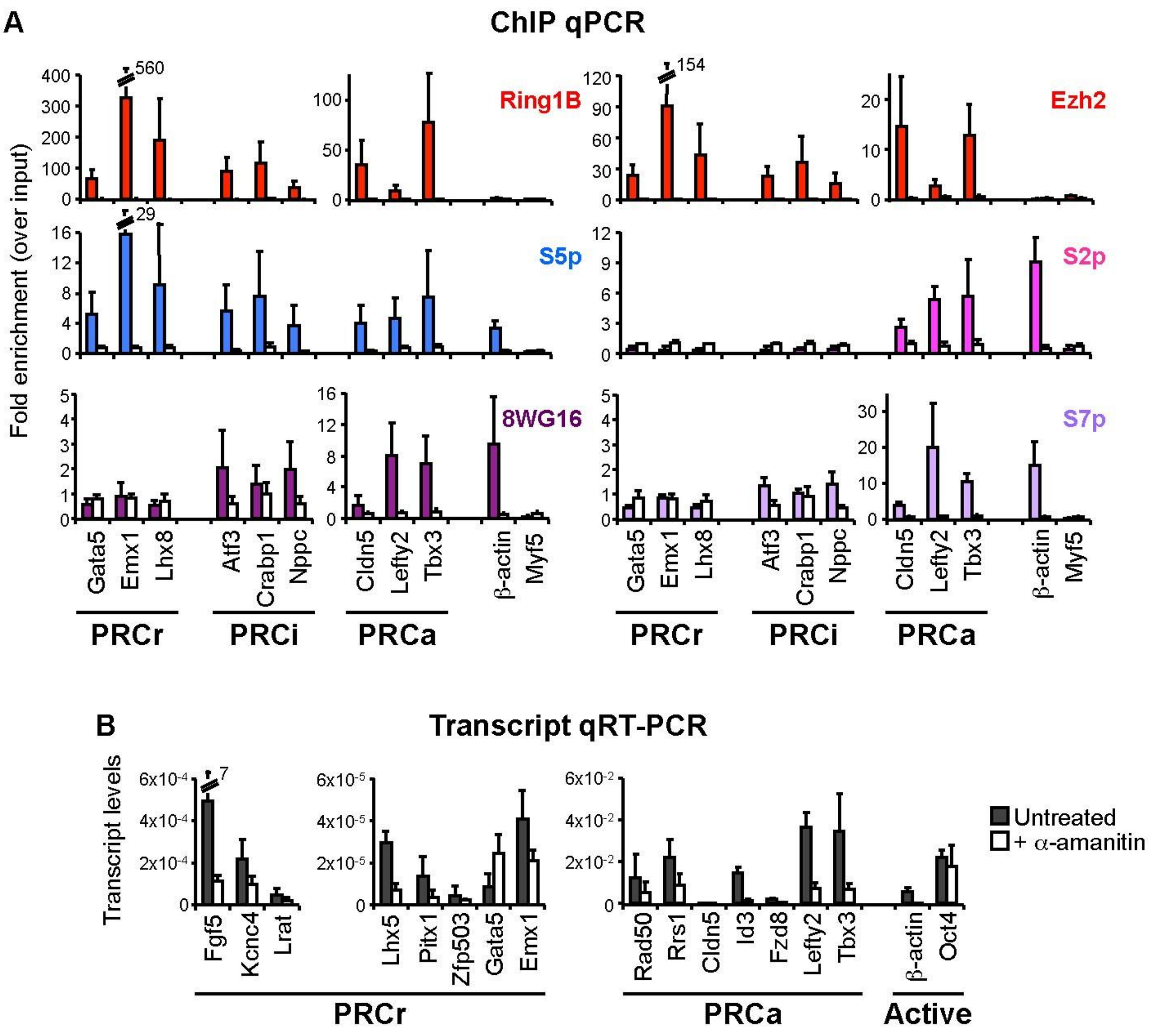
A
ChIP qPCR
Ring1B
Ezh2
S5p
S2p
8WG16
S7p
Fold enrichment (over input)
560
154
29
Gata5
Emx1
Lhx8
Atf3
Crabp1
Nppc
Cldn5
Lefty2
Tbx3
β-actin
Myf5
PRCr
PRCi
PRCa
B
Transcript qRT-PCR
Transcript levels
7
Fgf5
Kcnc4
Lrat
Lhx5
Pitx1
Zfp503
Gata5
Emx1
Rad50
Rrs1
Cldn5
Id3
Fzd8
Lefty2
Tbx3
β-actin
Oct4
PRCr
PRCa
Active
Untreated
+ α-amanitin

Supplementary Figure S4. Single gene analyses confirm the RNAPII modifications and expression status of genes from distinct PRC groups – related to Figure 4.

A, ChIP-qPCR analysis of representative genes from *PRCr*, *PRCi* and *PRCa* groups were analyzed for the markers shown, along with *Active* (*ß-actin*) and *Inactive* (*Myf5*) genes. Ring1B and Ezh2 were detected at PRC-targets but not at *Active* or *Inactive* genes. S5p was detected at PRC-target and *Active* genes, but not at *Myf5*. 8WG16 and S7p were absent from *PRCr* genes, detectable at low levels at *PRCi* genes, and at high levels at *PRCa* and *Active* genes. S2p was detected only at *PRCa* and *Active* genes. Background levels (mean enrichment from control antibodies and beads alone) shown as white bars. Mean and SD from 5 biological replicates.

B, Total RNA levels were measured using primers located at gene 5'-ends, and normalized to housekeeping genes. Open bars, alpha-amanitin treatment (7h, 75 µg/ml) to inhibit transcription. Transcripts are detected at all genes, shown by sensitivity to alpha-amanitin, but levels are 100-1000x higher at *PRCa* and *Active* than at *PRCr* genes, as expected. Mean and SD from 3 biological replicates.

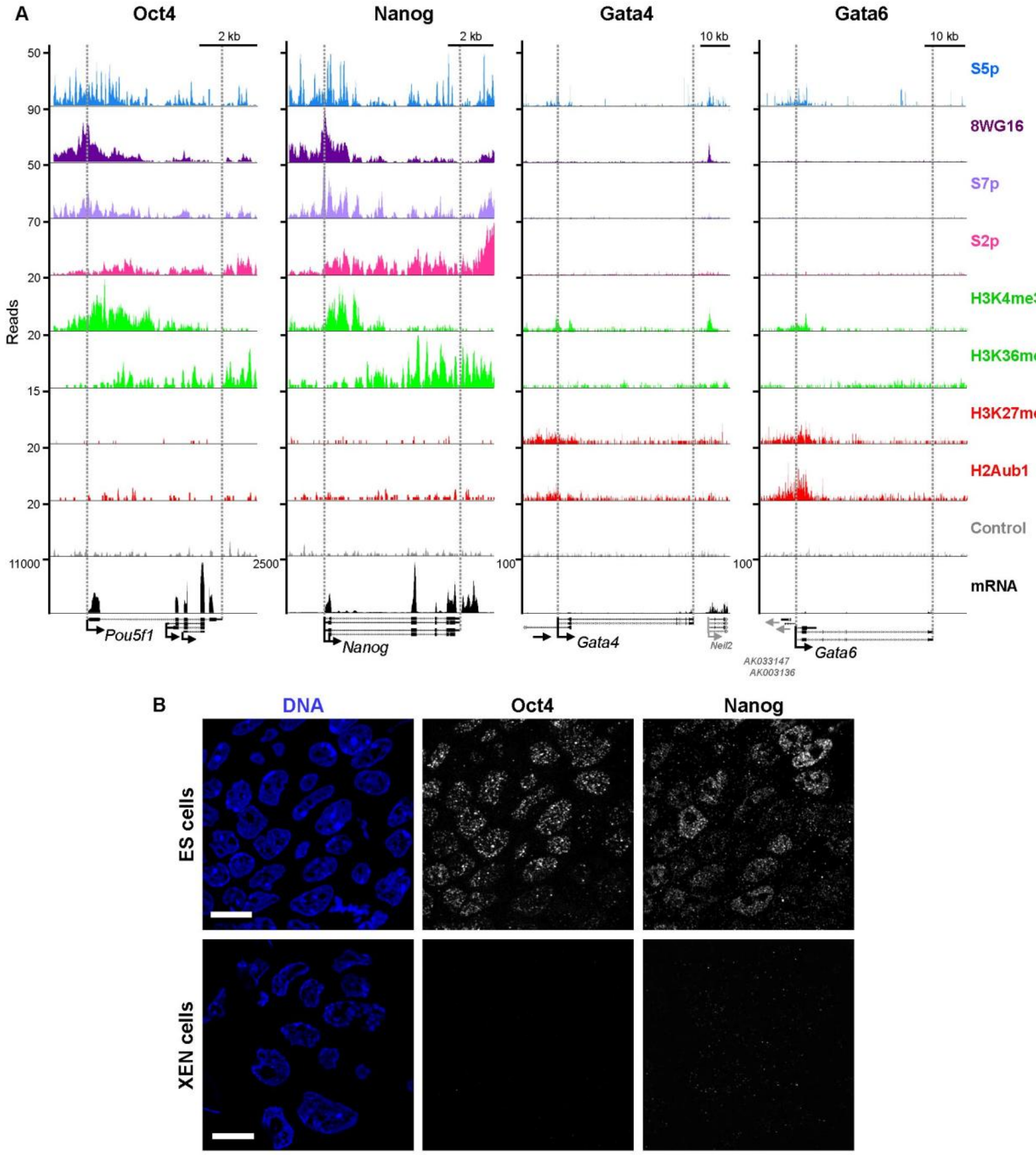

A
Oct4
Nanog
Gata4
Gata6
2 kb
10 kb
S5p
8WG16
S7p
S2p
H3K4me3
H3K36me3
H3K27me3
H2Aub1
Control
mRNA
Reads
Pou5f1
Nanog
Gata4
Neil2
Gata6
AK033147
AK003136
B
DNA
Oct4
Nanog
ES cells
XEN cells

Supplementary Figure S5. Active PRC-target genes do not represent ESC differentiation – related to Figure 5.

A, Pluripotency genes (*Oct4*, *Nanog*) show hallmarks of active genes, while early differentiation markers (*Gata4*, *Gata6*) show PRC-repression and no detectable activity (note difference in scale).

B, Immunofluorescence of Oct4 and Nanog in thin cryo-sections from ES and XEN cells demonstrates expression in all ESCs, albeit to variable extents, but not XEN cells. Labeling and image collection was performed in parallel in the same conditions and microscope settings. Bars, 10 µm.

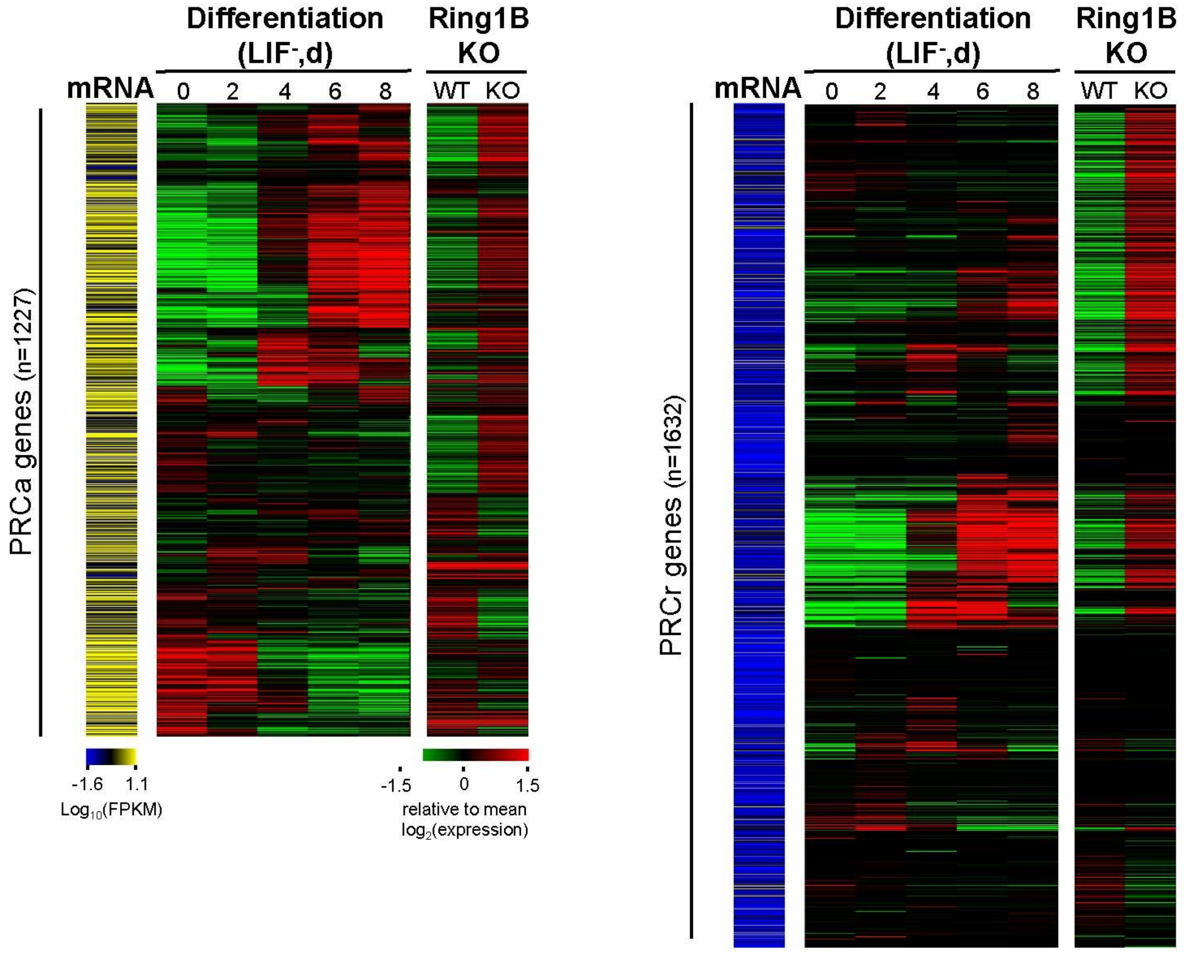
Differentiation (LIF-,d)
Ring1B KO
mRNA
0 2 4 6 8
WT KO
PRCa genes (n=1227)
-1.6 1.1
Log10(FPKM)
-1.5 0 1.5
relative to mean log2(expression)
PRCr genes (n=1632)

Supplementary Figure S6. *PRCa* and *PRCr* genes show de-repression upon *Ring1B* knockout but can be up- or down-regulated upon differentiation – related to Figure 6.
Analysis of microarray expression data for ESC differentiation (Shen et al., 2009) and *Ring1B*-knockout ES-ERT2 cells (Ku et al., 2008) shows that *PRCa* genes can be up- or down-regulated upon differentiation, but all show a tendency for up-regulation upon Ring1B depletion. *PRCr* genes show a similar percentage of genes de-repressed after *Ring1B* knockout, demonstrating the limits of the detection strategy. Red/green colors represent expression changes relative to the mean in this group of genes; absolute mRNA levels in ESCs (normalized to all RefSeq genes) are shown in the left hand panel. Genes ordered according to hierarchical clustering of differentiation microarray data.

Supplementary Table S1. ChIP-seq datasets used in this study. Libraries for new datasets were produced using magnetic beads, fragment size selection post-amplification, and sequenced at the Wellcome Trust Centre for Human Genetics (Oxford) or the MRC Clinical Sciences Centre (London), unless otherwise stated.
*Datasets used in downstream analyses (also colored); previously published datasets have reference and NCBI Gene Expression Omnibus Sample number (GSM) indicated.

ChIP-seq dataset	Antibody	Additional information	Cell type	Mapped reads (millions)
**RNAPII**				
**RNAPII-S5p** *	CTD4H8 (MMS-128P, Covance)	IP with magnetic beads.	ES-OS25	22
RNAPII-S5p	CTD4H8 (MMS-128P, Covance)	IP with sepharose beads.	ES-OS25	4
**RNAPII-8WG16** *	8WG16 (MMS-126R, Covance)		ES-OS25	24
**RNAPII-S7p** *	4E12 (Chapman *et al.*, 2007)		ES-OS25	11
**RNAPII-S2p** *	H5 (MMS-129R, Covance)	Fragment size selection post-amplification. Sequencing at WTCHG.	ES-OS25	33
RNAPII-S2p	H5 (MMS-129R, Covance)	Fragment size selection pre-amplification. Sequencing at BC Genomics.	ES-OS25	12
**PRC1**				
**Ring1B** *	A-20 (Atsuta *et al.*, 2001)		ES-OS25	28
**Ring1B** (GSM327669) *	A-20 (Atsuta *et al.*, 2001)	Data from (Ku *et al.*, 2008).	ES-V6.5	4
**H2Aub1** *	E6C5 (Upstate)		ES-OS25	18
**PRC2**				
**Ezh2** (GSM327668) *	39103, (Active Motif)	Data from (Ku *et al.*, 2008).	ES-V6.5	9
**Suz12** (GSM327667) *	ab12073 (Abcam)	Data from (Ku *et al.*, 2008).	ES-V6.5	13
**H3K27me3** (GSM307619) *	07-449 (Upstate)	Data from (Mikkelsen *et al.*, 2007).	ES-V6.5	8
**H3K27me3** (GSM686992-4) *	07-449 (Upstate)	Data from (Lienert *et al.*, 2011).	ES-129Sv-C57Bl/6	16
**Active histone modifications**				
**H3K4me3** (GSM307618) *	ab8580 (Abcam)	Data from (Mikkelsen *et al.*, 2007).	ES-V6.5	9
**H3K36me3** *	13C9 (Rechtsteiner *et al.*, 2010)		ES-OS25	23
H3K36me3 (GSM307620)	ab9050 (Abcam)	Data from (Mikkelsen *et al.*, 2007).	ES-V6.5	4
**Core histones**				
**H3** (GSM307624) *	ab1791 (Abcam)	Data from (Mikkelsen *et al.*, 2007).	ES-V6.5	7
**Control**				
**Mock IP** *		Beads only	ES-OS25	12

Supplementary Table S3. Enrichment in Gene Ontology (GO) terms at Active, PRC-target and Inactive clusters, related to Table 1.

GO terms associated with genes in each cluster were compared with terms present at other gene clusters or the whole group of RefSeq genes considered. GO term redundancy within the same ontology branch was trimmed to the highest term level. The most significantly enriched four terms are represented (unless fewer terms were enriched; hypergeometric test). Some terms are colored to aid data inspection.

Enriched at: / Relative to:	Inactive	PRCo	PRCr	PRCi	PRCa	Active
**Inactive** (n=5298)		localization (10^{-10})<br>developmental process (10^{-5})<br>regulation of biological quality (10^{-5})<br>locomotion (1x10^{-4})	developmental process (10^{-30})<br>metabolic process (10^{-30})<br>cellular process (10^{-30})<br>intracellular signaling pathway (10^{-30})	metabolic process (10^{-23})<br>localization (10^{-16})<br>developmental process (10^{-13})<br>cellular component organization (10^{-7})	metabolic process (10^{-30})<br>developmental process (10^{-30})<br>cellular component organization (10^{-24})<br>negative regulation of biological process (10^{-22})	metabolic process (10^{-30})<br>cellular component organization (10^{-30})<br>cellular process (10^{-30})<br>localization (10^{-23})
**PRCo** (n=798)	signaling (10^{-8})<br>multicellular organismal process (10^{-7})<br>response to pheromone (10^{-6})<br>biological regulation (0.03)		developmental process (10^{-26})<br>metabolic process (10^{-21})<br>cellular process (10^{-17})<br>biological regulation (10^{-11})	metabolic process (10^{-9})<br>intracellular signaling pathway (6x10^{-4})<br>macromolecule localization (9x10^{-4})<br>cellular process (0.006)	metabolic process (10^{-20})<br>cellular process (10^{-10})<br>developmental process (10^{-8})<br>intracellular signaling pathway (10^{-7})	metabolic process (10^{-30})<br>cellular process (10^{-11})<br>macromolecular localisation (10^{-9})<br>cellular component organisation (10^{-7})
**PRCr** (n=1632)	system process (10^{-30})<br>signaling (10^{-6})<br>oxidation reduction (2x10^{-4})<br>multi-organism process (0.002)	multi-organism process (10^{-6})<br>G-protein coupled receptor protein signaling pathway (10^{-6})<br>response to stimulus (0.002)<br>system process (0.004)		cytokine production (0.007)<br>macromolecule localization (0.007)<br>lipid metabolic process (0.007)<br>aromatic compound biosynthetic process (0.009)	macromolecule modification (3x10^{-4})<br>catabolic process (7x10^{-4})<br>protein metabolic process (8x10^{-4})<br>cell cycle (9x10^{-4})	organelle organization (10^{-13})<br>macromolecule localization (10^{-10})<br>cell cycle (10^{-10})<br>cell division (10^{-5})
**PRCi** (n=742)	signaling (10^{-12})<br>multicellular organismal process (10^{-11})<br>response to pheromone (10^{-6})<br>biological regulation (0.004)	system process (10^{-6})<br>signaling pathway (6x10^{-4})<br>immune system process (6x10^{-4})<br>anion transport (7x10^{-4})	developmental process(10^{-14})<br>multicellular organismal process (10^{-13})<br>biological regulation (10^{-13})<br>cellular process (10^{-7})		cellular process (8x10^{-4})<br>biological regulation (0.002)<br>growth (0.004)<br>developmental process (0.004)	metabolic process (10^{-7})<br>cellular process (7x10^{-4})<br>cellular component biogenesis (0.002)<br>cellular component organisation (0.007)
**PRCa** (n=1227)	signaling (10^{-15})<br>multicellular organismal process (10^{-10})<br>response to stimulus (0.01)	system process (10^{-9})<br>signaling pathway (10^{-6})<br>multi-organism process (3x10^{-4})<br>response to stimulus (6x10^{-4})	multicellular organismal process (10^{-12})<br>developmental process (10^{-9})<br>biological regulation (10^{-6})<br>nitrogen compound metabolic process (10^{-6})	behaviour (0.01)<br>localization (0.01)<br>response to drug (0.02)<br>aromatic compound biosynthetic process (0.02)		macromolecular localization (1x10^{-4})<br>organelle organisation (7x10^{-4})<br>cellular response to stress (0.002)<br>cellular localization (0.003)
**Active** (n=5707)	signaling (10^{-30})<br>multicellular organismal process (10^{-30})<br>biological regulation (10^{-22})<br>response to stimulus (10^{-20})	signaling (10^{-16})<br>multicellular organismal process (10^{-13})<br>response to stimulus (10^{-12})<br>ion transport (10^{-10})	multicellular organismal process (10^{-30})<br>developmental process (10^{-30})<br>biological regulation (10^{-30})<br>signalling (10^{-30})	signaling (10^{-9})<br>multicellular organismal process (10^{-7})<br>locomotion (2x10^{-4})<br>biological adhesion (3x10^{-4})	multicellular organismal process (10^{-18})<br>signaling (10^{-17})<br>developmental process (10^{-17})<br>biological regulation (10^{-12})	
**All** (n=15404)	signaling (10^{-30})<br>multicellular organismal process (10^{-30})<br>response to stimulus (10^{-11})<br>biological regulation (10^{-6})	multi-organism process (10^{-7})<br>response to stimulus (10^{-5})<br>immune system process (10^{-5})<br>locomotion (0.001)	developmental process (10^{-30})<br>biological regulation (10^{-30})<br>multicellular organismal process (10^{-30})<br>cellular process (10^{-16})	localization (10^{-6})<br>behaviour (4x10^{-4})<br>prosthetic group metabolic process (4x10^{-4})<br>aromatic compound biosynthetic process (0.001)	developmental process (10^{-15})<br>metabolic process (10^{-8})<br>intracellular signaling pathway (10^{-8})<br>growth (10^{-6})	metabolic process (10^{-30})<br>cellular component organization (10^{-23})<br>cellular process (10^{-19})<br>cellular component biogenesis (10^{-15})

Supplementary Methods

ES-OS25 cell culture

Mouse ES-OS25 cells (kindly donated by W. Bickmore) were grown on 0.1% gelatin-coated surfaces in GMEM-BHK21 supplemented with 10% fetal calf serum, 2 mM L-glutamine, 1% MEM non-essential amino acids (NEAA), 1 mM sodium pyruvate (all from Gibco, Invitrogen), 50 µM ß-mercaptoethanol, 1000 U/ml of human recombinant leukaemia inhibitory factor (LIF; Chemicon, Millipore, Chandler's Ford, UK) and 0.1 mg/ml Hygromycin (Roche) as described previously (Billon et al., 2002; Niwa et al., 2000).

Murine ES-ERT2 cell culture

Mouse ES-ERT2 ($Ring1A^{-/-}$; $Ring1B^{fl/fl}$; *Rosa26*::*CreERT2*) cells were a kind gift from M. Vidal and H. Koseki (Cales et al., 2008; Endoh et al., 2008). ES-ERT2 cells were cultured in 0.1% gelatin-coated flasks on mitomycin-inactivated SNLs as described previously (Stock et al., 2007). For *Ring1B* conditional deletion, ES-ERT2 cells were plated feeder-free on gelatin-coated plates 16h before supplementing the medium with 800 nM 4-hydroxy-tamoxifen (H7904, Sigma) for 24-72h.

Murine XEN cell culture

Murine XEN-IM8A1 cells (Kunath et al., 2005; kindly donated by J. Rossant) were cultured on 0.1% gelatin-coated surfaces in RPMI supplemented with 20% FCS, 2 mM L-glutamine, 1 mM sodium pyruvate, and 0.1 mM ß-mercaptoethanol, as described previously (Stock et al., 2007).

Drug treatments

To inhibit CDK9 and hence S2p, ES-OS25 cells were treated (1h) with 0.1-100 µM flavopiridol (from 50 mM stock in DMSO; a kind gift from Sanofi-Aventis, provided by Drug Synthesis and Chemistry Branch, Developmental Therapeutics Program, Division of Cancer Treatment and Diagnosis, National Cancer Institute, Bethesda, MD).
To inhibit RNAPII transcription, ES-OS25 cells were treated for 7h with 75 µg/ml alpha-amanitin (Sigma).

Chromatin immunoprecipitation

Fixed chromatin preparation

All markers, except H3K36me3 and H2Aub1, were mapped using fixed (cross-linked) chromatin. Fixed chromatin was prepared as described previously (Stock et al., 2007).

Native chromatin preparation

H3K36me3 and H2Aub1 were mapped in native (unfixed) chromatin, prepared as described previously (O'Neill and Turner, 2003; Szutorisz et al., 2005) with some modifications. Cells were washed in ice-cold PBS and incubated in 0.5% NP40 in 1X TBS (10 mM Tris-HCl pH 7.4, 3 mM $CaCl_2$, 2 mM $MgCl_2$) for 10 min. Cells were scraped and incubated on ice (50 min) to complete cell lysis. Nuclei were isolated by Dounce homogenization (60 strokes, 'Tight' pestle) and re-suspended in 25% sucrose in 1X TBS. 50% sucrose in 1X TBS was used to underlie this suspension,

before centrifugation. The nuclear pellet was washed once in 25% sucrose in 1X TBS and then re-suspended in 'digestion' buffer (50 mM Tris-HCl pH 7.4, 1 mM $CaCl_2$, 4 mM $MgCl_2$, 0.32 M sucrose) to a DNA concentration of 0.5 mg/ml (determined by measuring absorbance of alkaline-lysed chromatin).
Chromatin was digested (37°C, 10 min) with 2 U/ml micrococcal nuclease (Sigma) to produce fragments containing mainly mono- and di-nucleosomes. Digestion was stopped by addition of 5 mM EDTA on ice. The first supernatant (S1) was recovered, and the pellet re-suspended in 'lysis' buffer (1 mM Tris-HCl pH 7.4, 0.2 mM EDTA). After nuclear lysis was completed (30 min on ice and overnight at -20°C), a second supernatant (S2) was collected. After fragment size analysis, supernatants (S1 and S2) were combined. TBS, digestion and lysis buffers were supplemented with 5 mM NaF, 2 mM Na_3VO_4, 1 mM PMSF, and protease inhibitor cocktail.

Chromatin Immunoprecipitation (ChIP)

Arbitrary 'chromatin concentration' was obtained by measuring absorbance (280 nm) of alkaline-lysed chromatin (fixed or native), and using the conversion 50 mg/ml for 1 absorbance unit.
For mouse/rat IgG and IgM antibodies, protein-G-magnetic beads (Active Motif) were incubated with rabbit anti-mouse (IgG+IgM) or anti-IgM bridging antibodies, respectively (Jackson Immunoresearch; 10 µg per 50 µl beads) for 1h (4ºC) and washed with sonication buffer. For rabbit antibodies, magnetic beads were just washed with sonication buffer. Fixed (700 µg) or native (250 µg) chromatin was immunoprecipitated (4ºC, overnight) with 10-50 µg antibody and 50 µl beads (with/without bridging antibody).
Sepharose beads were used to produce an RNAPII-S5p ChIP-seq library for comparison; immunoprecipitation was performed according to (Stock et al., 2007).

ChIP washes and elutions

After immunoprecipitation using either IgG and IgM antibodies, beads were washed as described previously (Stock et al., 2007). Immune complexes were eluted from beads (65°C, 5 min; and room temperature, 15 min) with 50 mM Tris-HCl pH 8.0, 1 mM EDTA and 1% SDS. Elution was repeated and eluates pooled.

DNA purification, quantification and analysis

For fixed chromatin samples, reverse cross-linking was carried out (16h, 65°C) with addition of NaCl and RNase A. Native and fixed samples had EDTA increased to 5 mM and samples were incubated with 200 µg/ml proteinase K (50°C, 2h). DNA was recovered by phenol-chloroform extraction and ethanol precipitation. The final DNA concentration was determined by PicoGreen fluorimetry (Molecular Probes, Invitrogen) and samples were diluted to the same concentration (0.2 ng/µl). The same amount (0.5 ng) of immunoprecipitated and input DNA were analyzed by quantitative real-time PCR (qPCR). Amplifications (40 cycles) were performed using SensiMix NoRef (Quantace, London, UK) with DNA Engine Opticon 1/2 Real Time PCR system (BioRad, Hemel Hempstead, Hertfordshire, UK). Primer sequences (in 5' to 3' orientation) are listed below. Other primers were previously published (Stock et al., 2007).

***PRCr* genes**	
Fgf5 Promoter F	GAGCCAGCCCTGCAAGAT

Fgf5 Promoter R	GACGCTTCTCCCCGTGA
Fgf5 End F	CCGGAAAGAACATACATGATACA
Fgf5 End R	CAACTGCTTGTGTCACGTTAATT
Kcnc4 Promoter F	GGGTTCCACATCTGTTTCATC
Kcnc4 Promoter R	CCGGGTGTTTTTGCCTAT
Kcnc4 End F	TCAGCGTGGGCATAGTTG
Kcnc4 End R	AATGTTGAGCCGAAAGCG
Lrat Promoter F	AGAGCCAGGAGAGCAAGAGAT
Lrat Promoter R	CCAGGCACACTACCTCTTCA
Lrat End F	TGAAAACATCACCAGACATGACTTAG
Lrat End R	TGGCAAGTCGTGGATTTCTCT
Lhx5 Promoter F	GCCCTGACCGCAACG
Lhx5 Promoter R	TCCCCCACTTTCAAGCG
Lhx5 End F	ACCCCAGATTCACCGACATG
Lhx5 End R	TACGGCCGCTTCGTTGA
Zfp503 Promoter F	GTTGTCGACCTCGGTAGTGTTCC
Zfp503 Promoter R	GGCCTCTTCTCTTGAGCTATTCAACT
Zfp503 End F	CCGTCAGCGAGGAGTGAA
Zfp503 End R	CCTCGTGCGCTCATGATC
Pitx1 Promoter F	CAGATCAGCGTCGGACGATT
Pitx1 Promoter R	CACCACCACCGCACGAC
Pitx1 End F	CGCCCAGTTGTTGTAGGAGTAGC
Pitx1 End R	GGCTTCTCCAGGTCTGGTTCA
Emx1 Promoter F	ATCAGAGGAACCGCTCAGGC
Emx1 Promoter R	CATGGCCTCTGGGAACACC
Gata5 Promoter F	CGGTTCCCGAAGAGTGCAG
Gata5 Promoter R	CTCGCGCGGGGAAAA
Lhx8 Promoter F	TTTCCCTCCAGCTTACACACAAA
Lhx8 Promoter R	CTCTGACCCTCTGGAGCTGGTAT
***PRCi* genes**	
Atf3 Promoter F	CGGGTTAGCCGATTGG
Atf3 Promoter R	CAACGCGAGGGCTTTAA
Crabp1 Promoter F	AGGGAGTCAACGGAGTGATGAAT
Crabp1 Promoter R	TGTGATCTAGCACGAGATAAAGATGG
Nppc Promoter F	CCGAGGCTGGGAAATGAAC
Nppc Promoter R	CCGAAGGTGGGTGCTGTC
***PRCa* genes**	
Cldn5 F	CATCGTTGTCCGCGAGTTCTAT
Cldn5 R	AGTACTTGACCGGGAAGCTGAAC
Id3 F	CGGGTCGCACCAAATGA
Id3 R	ACACTGCCTCCACCCAGTTG
Lefty2 F	GGCTGTCCCCACACAGTG
Lefty2 R	CAGCATCCCCTACCTAGAGTCG
Rad50 F	GACCCAAAAGTTCCGAACAC
Rad50 R	GTGCGAGGCGGGAAT
Rrs1 F	CGGTTTAGGGTGCTGCTGTA
Rrs1 R	CTCCTTGTGCACCGTGATG
Tbx3 F	AACCCGAAGAAGACGTAGAAGATGA
Tbx3 R	ACTTACCTTCCTGACTTCGTGATGAC

Immunoprecipitation (IP) ‘cycle over threshold’ (Ct) values from the quantitative PCR (IP Ct) were subtracted from the input Ct values (Input Ct). This figure was converted into the fold enrichment using the equation $2^{(\text{input Ct} - \text{IP Ct})}$.

Sequential ChIP

Fixed chromatin was prepared and the first immunoprecipitations set up using magnetic beads as for single ChIP. ChIP washes were performed as for single ChIP. After the final wash in TE buffer, immune complexes were eluted from the beads twice (65°C, 5 min; and room temperature, 15 min) using only 40 µl of elution buffer (50 mM Tris pH 8.0, 1 mM EDTA and 1% SDS) per elution, giving a final eluate volume of 80 µl.

‘Re-ChIP dilution’ buffer (55 mM HEPES pH 7.9, 154 mM NaCl, 1.0 mM EDTA, 1.1% Triton X100, 0.11% Na-deoxycholate) was added to dilute the eluate 10-fold. Diluting the eluate 10-fold gives a final SDS concentration of 0.1% for optimal efficiency of the second immunoprecipitation, and adjusts all the components back to their concentration in sonication buffer (plus 5 mM Tris).

The second round of immunoprecipitations was set up following the standard protocol, adding magnetic beads and antibody directly. ChIP washes, elutions and DNA purification were carried out as in the standard protocol. Enrichment was calculated relative to the original input (thawed chromatin) using the same amount of DNA in the PCRs.

To test for contamination of antibody remaining from the first immunoprecipitation, we included a no-antibody control in the second round of immunoprecipitations as a negative control. As a positive control, we performed the first and second immunoprecipitations using the same antibody (Ring1B). The order of immunoprecipitations was also inverted to confirm specificity.

ChIP-seq library preparation

The quality of DNA used in the preparation of libraries for high-throughput sequencing was confirmed prior to library preparation by qPCR analyses of active, PRC-repressed and silent genes with previously characterized chromatin states (Stock et al., 2007).

ChIP-seq libraries were prepared from 10 ng DNA (quantified by PicoGreen and Qubit) according to Illumina protocols (Part #11257047 Rev A), with some modifications. Samples were PCR amplified prior to size selection on an agarose gel, enabling visualization of the amplified DNA fragments and therefore more careful extraction of appropriately sized fragments. After purification by QIAgen Gel Extraction, libraries were quantified by Qubit (Invitrogen) and qPCR, and library size was assessed by Bioanalyzer (Agilent). Fragment sizes were within the expected size distribution. The size range was 150-260bp (including adapters) for all libraries with the exception of RNAPII-S5p and -8WG16 where larger fragment sizes (250-500bp) were selected due to the distribution of these marks at promoters, where there is evidence that protection of DNA may occur (Ferrai et al., 2007).

For comparison, one library (RNAPII-S2p) was also produced by performing size selection prior to amplification (not shown).

RNA analysis

Gene expression analysis by Reverse Transcription PCR (RT-PCR)

Total RNA was prepared from ESCs (OS25 or ERT2) by TRIzol (Invitrogen) extraction using Phase Lock Gel tubes (5 Prime) following the manufacturer's instructions. Total RNA was immediately treated with TURBO DNase I (Ambion) according to the manufacturer's instruction. Treated RNA (1 µg) was reverse transcribed with 50 ng random primers and 10 U reverse transcriptase (Superscript II kit, Invitrogen) in a 20 µl reaction. The synthesized cDNA was diluted 1:10, and 2.5 µl used for qRT-PCR.
Results are normalized to *ß-actin*, *Ubc* and *G6PD* housekeeping genes (spliced transcripts detected using exon-exon primers). Transcripts were detected with primers that amplify sequences within the first exon (5' transcripts, detects both primary and spliced RNAs), other than for *Eno2* and *Hdac2*, where the first exons were too small, and so the amplicons extended into the first intron (detects primary transcripts). Primer sequences (in 5' to 3' orientation) are listed below. Other primers were previously published (Stock et al., 2007), or use the ChIP promoter sequences above.

**5' Expression**	
***PRCr* genes**	
Lhx5 F	CGCGACCCGTGACATG
Lhx5 R	GTGGCTCTCAGCCTTCCTAGTCT
***PRCa* genes**	
Fzd8 F	CTGCCACAACCCCTTCTTTA
Fzd8 R	AAAGCGCTCCATATCGATGA
Lefty2 F	CGATGACCGAGGAACAGG
Lefty2 R	AATTCTGGCTGAACCTCTTGC
Hmg2a F	GGTTGCTCTCTCCGCAAAG
Hmg2a R	TTCTTTCCCCGCCTAACATT
S100a6 F	CGTTAGGGCCCGCTAAA
S100a6 R	CAGCGCCTGAGGCAAG
Tpm4 F	GGAGCCCAGCAGAACGATT
Tpm4 R	TTGCGCGCGATCCTCT
Hk1 F	CAGTAGCCCTGGTGAGCAGT
Hk1 R	TGTAGCCAATGGGGACTGAG
Eno2 F	GAGGAGGTGACAGCCAGAAA
Eno2 R	GTCTGCAGTCCTCGAGGTGA
Hdac2 F	CGGCAAGAAGAAAGTGTGCT
Hdac2 R	CACCCTAAACCTGCGTGTG

mRNA-seq library preparation

Total RNA was prepared from ES-OS25 cells by TRIzol extraction using Phase Lock Gel tubes and 8 µg was prepared for mRNA sequencing. The mRNA-seq library was prepared according to Illumina's instructions (#1004898 Rev A) with some modifications. After selection of polyA-containing mRNA molecules, RNA was further depleted for ribosomal RNA (rRNA) using the Ribominus kit (Invitrogen), according to the manufacturer's instructions. The Bioanalyzer validated the loss of rRNA and appropriate size distribution of fragments prior to library generation. The

library was quantified by Qubit, and library size was assessed by Bioanalyzer before high-throughput sequencing.

High-throughput sequencing

Libraries were sequenced using Illumina Sequencing Technology at the WTCHG Genomics facility or the MRC-CSC Genomics laboratory using an Illumina Genome Analyzer II, according to the manufacturer's instructions.

Analysis of genome-wide data

ChIP-seq datasets

A summary of ChIP-seq datasets is presented in Supplementary Table S1.

mRNA-seq and microarray datasets used for gene expression analyses

We generated mRNA-seq data after polyA$^+$ RNA purification and ribosomal RNA depletion. Published mRNA-seq data was obtained from (Cloonan et al., 2008). Gene expression data for mouse ES-V6.5 cells were obtained from previously published gene lists (Mikkelsen et al., 2007).
Affymetrix microarray mRNA profiles for mouse ES-ERT2 cells prior to and 48h after *Ring1B* deletion (Ring1A/B-dKO) were obtained from published CEL files (GSM265042, GSM265043; Endoh et al., 2008).
Raw Affymetrix microarray expression datasets for mouse ESCs in undifferentiated and differentiated (2, 4, 6, and 8 days after LIF withdrawal) states were obtained from a published CEL file (GSE19165; Shen et al., 2009). Raw Affymetrix data was normalized and annotated using the GCRMA (Gentleman et al., 2004; Wu et al., 2004) and mouse4302.db Bioconductor (Gentleman et al., 2004) packages, respectively. To visualize differentiation microarray data (Fig. 6C, Supplementary S6), we used MultiExperiment Viewer (Saeed et al., 2006; Saeed et al., 2003).

Aligning next-generation sequencing reads to the genome

Sequenced reads (36–72bp) from our ChIP-seq libraries were aligned against the UCSC mouse mm9 genome (July 2007) using Illumina Eland extended software. Reads from our mRNA-seq library (38bp) were aligned to the mm9 genome and UCSC annotated transcripts (UCSC Known Gene annotations) using Tophat (Trapnell et al., 2009) version 1.0.13 and Cufflinks (Trapnell et al., 2010) version 0.8.2, to allow detection of reads crossing exon-exon junctions and calculation of FPKM levels per transcript. Published ChIP-seq datasets were re-mapped to the mm9 genome using Bowtie (Langmead et al., 2009) software version 0.9.8.1 (allowing 2 mismatches and unique reads only, as for alignments in Illumina software), to allow direct comparison with our data.

Creating a non-redundant gene list

From the RefSeq universe, we wished to create a non-redundant list of genes to analyze. We relied upon UCSC Known Gene annotations (mm9 version) and their associated RefSeq IDs to build our initial candidate gene set. Genes belonging to 'random chromosomes' were discarded. To link gene transcripts into clusters that represent genes, we used information from *mm9.KnownIsoforms.clusterid* table. The initial dataset comprised a total of 18,860 gene clusters, each represented by one or more RefSeq genes. We selected the 'representative' UCSC gene from each cluster according to the following criteria:

1. If TSS co-ordinates differ, select the gene with the highest RNAPII-S5p in a 2kb window centred at the TSS,
2. If ambiguity still exists and if TES co-ordinates differ, take the gene with the highest RNAPII-S2p in a 4kb window centred at the TES,
3. If ambiguity still exists, select the canonical gene as annotated in the *mm9.knownCanonical* UCSC table. According to this process, the gene with the highest number of coding bases is chosen as the representative gene, and
4. Finally, for complete duplicate entries (identical co-ordinates and ID), select one gene arbitrarily.

This process produced a non-redundant list of 18,860 UCSC Known Genes, and their corresponding RefSeq IDs.

Gene binary classifications

To investigate the combinatorial association of each gene with RNAPII modifications or PRC, we classified each gene as positive or negative for each marker at regions of interest (Hebenstreit et al., 2011). We integrated the total number of reads within the regions of interest for each marker across all RefSeq genes. To convert continuous values to binary classifications, we used an expectation maximization algorithm (*optim* function in R) to fit a two-component mixture distribution, with the curve on the left representing background from the IP and the distribution on the right representing the specific IP signal. We tested the distribution of the signal from mock immunoprecipitation ChIP-seq data (control) and found that it displays a Normal distribution. The distribution of background in the ChIP datasets was modelled with a Truncated Normal, as sequencing depth was not sufficient to cover all background reads. The specific IP signal on the right was best modelled with a lognormal curve that fits the long tails observed. The advantage of this approach is the derivation of a probabilistic criterion for identifying a threshold at a specific false discovery rate (FDR). The FDR is the quotient between the expected proportion of false positive detections and the total number of detections.
To identify the threshold of positive detection for each marker, we used an FDR of <1%, thus setting the threshold to a value where the proportion of ‘noise’ corresponds to less than 1% of the total area of the mixture on the right of the threshold, ensuring that the number of false positive detections will be small. The threshold was then used to categorize the continuous values into binary ‘positive’ or ‘negative’ ([1,0]) events that denote, respectively, the presence or absence of a given histone mark or RNAPII (either at TSS or 3’-end regions). Gene classifications are provided in Supplementary Table S2.

Removing overlapping windows

Due to the arrangement of genes within the genome and the extension of RNAPII beyond the 3’-end of genes, the windows of interest for two genes can overlap. If both of these windows are classified as ‘positive’, it is not possible to declare to which gene the positive signal belongs. Therefore, we removed genes where windows positive for a given marker overlap by more than 10% (200bp). This produced a list of 15,404 non-overlapping RefSeq genes.

Computing average profiles, boxplots, correlations and heatmaps

Average marker enrichment within regions of interest (*e.g.* 2kb centred on TSS or 2kb downstream of TES) was plotted for each ChIP-Seq dataset. Read ‘depth of

coverage' was computed by calculating the area under the coverage curve (*i.e.* number of sequenced nucleotides contained within the window of interest).
To produce average ChIP-seq profiles (*e.g.* Fig. 1C) and heatmaps (Supplementary Fig. S2C), depth of coverage was calculated for non-overlapping windows of 10 or 100bp, respectively, covering each region of interest.
For mRNA-seq data, depth within exonic regions was calculated for non-overlapping windows each covering 1% of the total exonic region length; counts were then normalized to the total number of bases in each window. Exon coordinates for UCSC Known Genes were downloaded from the UCSC table browser.
Boxplots displaying the median, upper and lower quartiles and the range of the data were generated by calculating the depth of coverage in windows of interest (*e.g.* Fig. 1D). Log_{10} transformation was applied before plotting heatmaps, boxplots and correlations. As values equal to zero are meaningful in ChIP-seq and mRNA-seq data, a pseudo-count of 1 or 0.0001 was added prior to the logarithm transformation for ChIP-seq or mRNA-seq FPKM levels, respectively, unless otherwise stated.

CpG content calculation

DNA sequences covering -1 to +1kb of the TSS of each gene were downloaded from the UCSC table browser. CpG percentage was calculated as the ratio between the number of CpG dinucleotides (x2) and the region length.

CpG O/E calculation and distribution pattern

CpG observed over expected ratio (O/E) was calculated in 200bp windows with an inter-window overlap of 150bp. To produce average CpG O/E profiles, genes were aligned according to their TSSs, and the mean CpG O/E value at each window position was calculated, within a region covering -3 to +3kb of each TSS.

Hierarchical clustering

The input matrix was composed of 15,404 RefSeq genes and 7 binary variables [S5p (± 1kb TSS), 8WG16 (TSS), S7p (TSS), S5p (2kb downstream TES), S2p (2kb upstream TES), H3K27me3 (TSS) and H2Aub1 (TSS)]. All pairwise dissimilarities in the data matrix were computed in R statistical software using the Gower coefficient; hierarchical clustering was calculated using the average linkage method and the function *hclust* in R.

Gene ontology (GO) analysis

Analysis of functional enrichment was performed using the Fisher's exact test implemented in the topGO Bioconductor package (Alexa et al., 2006) and the GO term to Entrez gene ID annotation provided within the org.Mm.eg.db Bioconductor annotation database (version 2.4.1; Gentleman et al., 2004). Enrichment of GO terms for each gene cluster was tested using all genes within every cluster as a reference set, whereas for direct comparisons between clusters of interest, the union of those clusters was used as a reference set. Significance was identified for GO terms with p-values less than 0.05 and, in order to capture GO terms of higher order annotation, the resulting enriched GO terms were filtered to their significant ancestors which were highest within the GO graph.

KEGG analysis

Annotation for KEGG (Kyoto Encyclopedia of Genes and Genomes) pathways and their associated genes were retrieved from the KEGG FTP site

(ftp://ftp.genome.jp/pub/kegg/; Kanehisa and Goto, 2000). Enrichment of KEGG pathways by group members was assessed by hypergeometric testing using the R Stats package (R Development Core Team, 2008) and false discovery rates calculated using the R Multtest package (R Development Core Team, 2008).

Immunofluorescence detection of pluripotency markers

Immunofluorescence of Oct4 and Nanog was performed on fixed whole cells or cryosections of ESCs and XEN cells, essentially as described previously (Guillot et al., 2004; Xie et al., 2006).
Briefly, cells were fixed with 4% PFA in 250 mM HEPES (pH 7.6) and either permeabilized and incubated with antibodies (Fig. 1A) or embedded in sucrose solution and frozen in liquid nitrogen, before sectioning and immunolabeling (sections ~ 200nm thick; Fig. 5C bottom panels; Supplementary S5B). To maximise detection of Nanog, and prevent steric hindrance due to antibody competition, whole cells or thin cryosections were sequentially immunolabelled with primary antibodies against Nanog (2h; 1:50; Abcam ab80892), AlexaFluor488-conjugated donkey antibodies against rabbit Ig (1h; 1:1000; Invitrogen), Oct4 (2h; 1:100; BD BioSciences 611202) and AlexaFluor555-conjugated donkey antibodies against mouse IgG (1h; 1:1000; Invitrogen). Nuclei were stained (45 min) with DAPI before mounting in VectaShield and imaging.

Cryo-DNA-FISH combined with RNAPII, Ezh2 or Nanog immunofluorescence

Cryo-immunofluorescence detection of RNAPII-S5p, RNAPII-S2p, Nanog or Ezh2, combined with DNA fluorescence *in situ* hybridization (FISH) on ultrathin cryosections (immuno-cryoFISH) was performed essentially as described before (Branco and Pombo, 2006; Ferrai et al., 2010).
Briefly, for Fig. 2D, ultrathin cryosections (~140-150 nm thick) were first immuno-labelled with murine primary antibodies specifically against RNAPII phosphorylated on S2 (2h; H5; IgM, 1:2000; Covance) or S5 (2h; CTD4H8; IgG,1:3000; Covance), and detected with AlexaFluor488-conjugated donkey antibodies against mouse Ig (1h; 1:1000; Invitrogen, UK). The specificity of antibodies to phosphorylated RNAPII was tested by treatment with alkaline phosphatase (2h; 37°C; 0.5 U/µl; New England Biolabs, Hitchin, UK) to show non-detectable RNAPII signals in western blot (not shown) and immunofluorescence (Ferrai et al., 2010).
For Fig. 5C, cryoFISH panels, cryosections were sequentially incubated with (a) CTD4H8, H5 (as above) or with mouse antibody against Ezh2 (2h, 1:50, BD BioSciences 612667), (b) AlexaFluor680-conjugated donkey antibodies against mouse Ig (1h; 1:500; Invitrogen), (c) rabbit anti-Nanog (2h; 1:100), and (d) AlexaFluor488-conjugated donkey antibodies against rabbit Ig (1h; 1:1000). Sequential immunolabelling was chosen for optimal detection of RNAPII or Ezh2. After immunolabelling, cryosections were fixed with 8% PFA in PBS (2h), prior to DNA-FISH, to preserve immunocomplexes before FISH. Immuno-cryoFISH was performed as previously described (Branco and Pombo, 2006; Ferrai et al., 2010). For Fig. 2D, loci were detected using BAC probes as follow: *ß-actin* (RP23-11E16); *Atoh1* (RP24-77K22); *Nkx2-2* (RP24-555M6); *Msx1* (RP24-463H20); *HoxA7* (RP24-466A1) and *Myf5* (RP24-28O11). For Fig. 5C, *Lefty2* locus was detected with fosmid probe (G135P67408A6). BAC and fosmid probes were obtained from BACPAC Resources (California, USA). Probes were labelled with tetramethyl-

rhodamine-5-dUTP or digoxigenin-11-dUTP by nick translation (Roche), and purified using MicroBioSpin P-30 chromatography columns (BioRad). The signal of rhodamine-labelled probes was amplified with rabbit anti-rhodamine antibodies (2h; 1:500; Invitrogen) and Cyanine3-conjugated donkey antibodies against rabbit IgG (1h; 1:1000; Jackson ImmunoResearch Laboratories). Probes labelled with digoxigenin were detected with sheep anti-digoxigenin Fab fragments (2h; 1:200; Roche) and AlexaFluor555 donkey antibodies against sheep IgG (1h; 1:1000; Invitrogen). Nuclei were stained with DAPI and coverslips were mounted with VectaShield before imaging.

Microscopy and Image Analysis

Images were acquired on a confocal laser-scanning microscope (Leica TCS SP5; 60x oil objective, NA 1.4) equipped with a 405 nm diode, and Argon (488 nm), HeNe (543 nm) and HeNe (633 nm) lasers, using pinhole equivalent to 1 Airy disk. Images from different channels were collected sequentially to prevent fluorescence bleedthrough. Raw images (TIFF files) were merged in ImageJ and contrast stretched without thresholding in Adobe Photoshop. Association of DNA loci with RNAPII (S2p or S5p) or Ezh2 was scored as ‘co-localized’ (signals overlap by ≥1 pixel) or ‘separated’ (Ferrai et al., 2010).

RNAPII western blotting

Whole cell extracts for RNAPII westerns were prepared by lysing cells in ice-cold ‘lysis’ buffer (Daniel and Carling, 2002), scraping, and shearing DNA by passage through a 25G needle. Cell lysates (0.5 µg total protein for 4H8, 10 µg for 4E12, 5 µg for all other RNAPII antibodies) were resolved on 7.5% Tris-HCl or 3-8% Tris-acetate SDS-PAGE gels. Membranes were blocked (1h), incubated (2h) with primary antibody, washed, and incubated (1h) with HRP-conjugated secondary antibodies, all in blocking buffer (10 mM Tris-HCl, 150 mM NaCl, 0.1% Tween-20, pH 8.0; 5% non-fat dry milk). Membranes were washed (30 min) in blocking buffer without milk and briefly in 0.1% Tween-20 in PBS. HRP-conjugated antibodies were detected with ECL western blotting detection reagents (Amersham).
To dephosphorylate RPB1, membranes were pre-incubated (37°C, 1h) in 0.1 U/µl alkaline phosphatase. Lack of antibody binding proves specificity for phospho-epitopes.
For westerns using RPB1 replacement mutants, protein samples were harvested using Laemmli buffer, 48h after induction by washing out tetracyclin from medium following alpha-amanitin (2 µg/ml) treatment. Protein from 200,000 cells was loaded per lane, and subjected to SDS-PAGE on 6% gels. After incubation with primary antibody in blocking buffer overnight, membranes were stained with affinity purified, IR-labelled secondary antibodies against rat (680nm; Alexa, Invitrogen) and mouse (800nm; Rockford, Biomol), and revealed using the Odyssey (Licor).

Enzyme-linked immunoabsorbant assays (ELISA)

Specificity of RNAPII modification antibodies was analyzed by ELISA using CTD peptides containing different modifications (not shown). S5p antibodies 4H8, 3E8 and ab5131 react only with S5p peptides. 3E8 and ab5131 binding is inhibited by S2p in the same repeat, whereas 4H8 binding is not. Antibody 8WG16 binding is inhibited by double phosphorylation and by single S2p, S7p or Y1p in shorter CTD peptides. Antibodies H5 and 3E10 both detect S2p peptides, but H5 binding is less

sensitive to additional phosphorylation. 4E12 binding is specific for S7p peptides and is sensitive to additional CTD modifications.
Antigens (CTD-1 to CTD-12) were coupled (1h, 37°C) to maleimide plates in carbonate buffer (pH 9.5) and blocked (30 min) with PBS/milk (1%). Primary antibodies were added to the wells and incubated (30 min). After an additional washing and blocking step, biotin-coupled antibodies recognizing the IgG subclasses of the primary antibodies, were added and incubated (30 min). Following another washing and blocking step, peroxidase attached to avidin was added to the wells. After washing (5x) in PBS, 50 µl of substrate buffer (containing o-phenylenediamine and H_2O_2; pH 5.0) were added and after change of color, samples were measured at 405 nm in an ELISA-reader.
Antigens (CTD, CTDa to CTDi) were coated on microtiter plates and incubated with decreasing concentrations of each antibody. After incubation with peroxidase-conjugated secondary antibody and washing, the colorimetric signal of tetramethylbenzidine was measured (absorbance 405 nm) using a plate reader.

Peptide	Sequence (red=phospho)	Phospho-site
CTD-1	YSPTSPSYSPTSPSC	-
CTD-2	YSP**T**SPSYSPTSPSC	T4
CTD-3	YSP**TS**PSYSPTSPSC	T4, S5
CTD-4	YSPT**S**PSYSPTSPSC	S5
CTD-5	YSPTSP**S**YSPTSPSC	S7
CTD-6	YSPTSPS**Y**SPTSPSC	Y1
CTD-7	YSPTSPSY**S**PTSPSC	S2
CTD-8	YSPT**S**PSY**S**PTSPSC	S5, S2
CTD-9	SPSY**S**PT**S**PSYSPTC	S2, S5
CTD-10	YSPTSPS**YS**PTSPSC	Y1, S2
CTD-11	YSPT**S**P**S**YSPTSPSC	S5, S7
CTD-12	YSPTSP**S**Y**S**PTSPSC	S7, S2
CTD	SYSPTSPSYSPTSPSYSPC	-
CTD-a	SYSPT**S**PSYSPTSPSYSPC	S5
CTD-b	SYSPTSP**S**YSPTSPSYSPC	S7
CTD-c	SYSPTSPSY**S**PTSPSYSPC	S2
CTD-d	SYSPT**S**P**S**YSPTSPSYSPC	S5, S7
CTD-e	SYSPT**S**PSY**S**PTSPSYSPC	S5, S2
CTD-f	SYSPTSP**S**Y**S**PTSPSYSPC	S7, S2
CTD-g	SYSPTSP**S**YSPT**S**PSYSPC	S7, S5
CTD-h	SYSPTSPSY**S**PT**S**PSYSPC	S2, S5
CTD-i	SYSPTSPSY**S**PTSP**S**YSPC	S2, S7

Supplementary References

Alexa, A., Rahnenfuhrer, J., and Lengauer, T. (2006). Improved scoring of functional groups from gene expression data by decorrelating GO graph structure. Bioinformatics *22*, 1600-1607.

Billon, N., Jolicoeur, C., Ying, Q.L., Smith, A., and Raff, M. (2002). Normal timing of oligodendrocyte development from genetically engineered, lineage-selectable mouse ES cells. J Cell Sci *115*, 3657-3665.

Branco, M.R., and Pombo, A. (2006). Intermingling of chromosome territories in interphase suggests role in translocations and transcription-dependent associations. PLoS Biol *4*, e138.

Cales, C., Roman-Trufero, M., Pavon, L., Serrano, I., Melgar, T., Endoh, M., Perez, C., Koseki, H., and Vidal, M. (2008). Inactivation of the polycomb group protein Ring1B unveils an antiproliferative role in hematopoietic cell expansion and cooperation with tumorigenesis associated with Ink4a deletion. Mol Cell Biol *28*, 1018-1028.

Carninci, P., Sandelin, A., Lenhard, B., Katayama, S., Shimokawa, K., Ponjavic, J., Semple, C.A., Taylor, M.S., Engstrom, P.G., Frith, M.C.*, et al.* (2006). Genome-wide analysis of mammalian promoter architecture and evolution. Nat Genet *38*, 626-635.

Chapman, R.D., Heidemann, M., Albert, T.K., Mailhammer, R., Flatley, A., Meisterernst, M., Kremmer, E., and Eick, D. (2007). Transcribing RNA polymerase II is phosphorylated at CTD residue serine-7. Science *318*, 1780-1782.

Cloonan, N., Forrest, A.R., Kolle, G., Gardiner, B.B., Faulkner, G.J., Brown, M.K., Taylor, D.F., Steptoe, A.L., Wani, S., Bethel, G.*, et al.* (2008). Stem cell transcriptome profiling via massive-scale mRNA sequencing. Nat Methods *5*, 613-619.

Daniel, T., and Carling, D. (2002). Functional analysis of mutations in the gamma 2 subunit of AMP-activated protein kinase associated with cardiac hypertrophy and Wolff-Parkinson-White syndrome. J Biol Chem *277*, 51017-51024.

Endoh, M., Endo, T.A., Endoh, T., Fujimura, Y., Ohara, O., Toyoda, T., Otte, A.P., Okano, M., Brockdorff, N., Vidal, M.*, et al.* (2008). Polycomb group proteins Ring1A/B are functionally linked to the core transcriptional regulatory circuitry to maintain ES cell identity. Development *135*, 1513-1524.

Ferrai, C., Munari, D., Luraghi, P., Pecciarini, L., Cangi, M.G., Doglioni, C., Blasi, F., and Crippa, M.P. (2007). A transcription-dependent micrococcal nuclease-resistant fragment of the urokinase-type plasminogen activator promoter interacts with the enhancer. J Biol Chem *282*, 12537-12546.

Ferrai, C., Xie, S.Q., Luraghi, P., Munari, D., Ramirez, F., Branco, M.R., Pombo, A., and Crippa, M.P. (2010). Poised transcription factories prime silent uPA gene prior to activation. PLoS Biol *8*, e1000270.

Gentleman, R.C., Carey, V.J., Bates, D.M., Bolstad, B., Dettling, M., Dudoit, S., Ellis, B., Gautier, L., Ge, Y., Gentry, J.*, et al.* (2004). Bioconductor: open software development for computational biology and bioinformatics. Genome Biol *5*, R80.

Guillot, P.V., Xie, S.Q., Hollinshead, M., and Pombo, A. (2004). Fixation-induced redistribution of hyperphosphorylated RNA polymerase II in the nucleus of human cells. Exp Cell Res *295*, 460-468.

Hebenstreit, D., Gu, M., Haider, S., Turner, D.J., Lio, P., and Teichmann, S.A. (2011). EpiChIP: gene-by-gene quantification of epigenetic modification levels. Nucleic Acids Res *39*, e27.

Kanehisa, M., and Goto, S. (2000). KEGG: kyoto encyclopedia of genes and genomes. Nucleic Acids Res *28*, 27-30.

Kent, W.J., Zweig, A.S., Barber, G., Hinrichs, A.S., and Karolchik, D. (2010). BigWig and BigBed: enabling browsing of large distributed datasets. Bioinformatics *26*, 2204-2207.

Ku, M., Koche, R.P., Rheinbay, E., Mendenhall, E.M., Endoh, M., Mikkelsen, T.S., Presser, A., Nusbaum, C., Xie, X., Chi, A.S.*, et al.* (2008). Genomewide analysis of PRC1 and PRC2 occupancy identifies two classes of bivalent domains. PLoS Genet *4*, e1000242.

Kunath, T., Arnaud, D., Uy, G.D., Okamoto, I., Chureau, C., Yamanaka, Y., Heard, E., Gardner, R.L., Avner, P., and Rossant, J. (2005). Imprinted X-inactivation in extra-embryonic endoderm cell lines from mouse blastocysts. Development *132*, 1649-1661.

Langmead, B., Trapnell, C., Pop, M., and Salzberg, S.L. (2009). Ultrafast and memory-efficient alignment of short DNA sequences to the human genome. Genome Biol *10*, R25.

Lienert, F., Mohn, F., Tiwari, V.K., Baubec, T., Roloff, T.C., Gaidatzis, D., Stadler, M.B., and Schubeler, D. (2011). Genomic prevalence of heterochromatic H3K9me2 and transcription do not discriminate pluripotent from terminally differentiated cells. PLoS Genet *7*, e1002090.

Mikkelsen, T.S., Ku, M., Jaffe, D.B., Issac, B., Lieberman, E., Giannoukos, G., Alvarez, P., Brockman, W., Kim, T.K., Koche, R.P.*, et al.* (2007). Genome-wide maps of chromatin state in pluripotent and lineage-committed cells. Nature *448*, 553-560.

Niwa, H., Miyazaki, J., and Smith, A.G. (2000). Quantitative expression of Oct-3/4 defines differentiation, dedifferentiation or self-renewal of ES cells. Nat Genet *24*, 372-376.

O'Neill, L.P., and Turner, B.M. (2003). Immunoprecipitation of native chromatin: NChIP. Methods *31*, 76-82.

R Development Core Team (2008). R: A language and environment for statistical computing. ISBN 3-900051-07-0 http://www.R-project.org.

Saeed, A.I., Bhagabati, N.K., Braisted, J.C., Liang, W., Sharov, V., Howe, E.A., Li, J., Thiagarajan, M., White, J.A., and Quackenbush, J. (2006). TM4 microarray software suite. Methods Enzymol *411*, 134-193.

Saeed, A.I., Sharov, V., White, J., Li, J., Liang, W., Bhagabati, N., Braisted, J., Klapa, M., Currier, T., Thiagarajan, M.*, et al.* (2003). TM4: a free, open-source system for microarray data management and analysis. Biotechniques *34*, 374-378.

Shen, X., Kim, W., Fujiwara, Y., Simon, M.D., Liu, Y., Mysliwiec, M.R., Yuan, G.C., Lee, Y., and Orkin, S.H. (2009). Jumonji modulates polycomb activity and self-renewal versus differentiation of stem cells. Cell *139*, 1303-1314.

Stock, J.K., Giadrossi, S., Casanova, M., Brookes, E., Vidal, M., Koseki, H., Brockdorff, N., Fisher, A.G., and Pombo, A. (2007). Ring1-mediated ubiquitination of H2A restrains poised RNA polymerase II at bivalent genes in mouse ES cells. Nat Cell Biol *9*, 1428-1435.

Szutorisz, H., Canzonetta, C., Georgiou, A., Chow, C.M., Tora, L., and Dillon, N. (2005). Formation of an active tissue-specific chromatin domain initiated by epigenetic marking at the embryonic stem cell stage. Mol Cell Biol *25*, 1804-1820.

Trapnell, C., Pachter, L., and Salzberg, S.L. (2009). TopHat: discovering splice junctions with RNA-Seq. Bioinformatics *25*, 1105-1111.

Trapnell, C., Williams, B.A., Pertea, G., Mortazavi, A., Kwan, G., van Baren, M.J., Salzberg, S.L., Wold, B.J., and Pachter, L. (2010). Transcript assembly and quantification by RNA-Seq reveals unannotated transcripts and isoform switching during cell differentiation. Nat Biotechnol *28*, 511-515.

Valen, E., Pascarella, G., Chalk, A., Maeda, N., Kojima, M., Kawazu, C., Murata, M., Nishiyori, H., Lazarevic, D., Motti, D.*, et al.* (2009). Genome-wide detection and analysis of hippocampus core promoters using DeepCAGE. Genome Res *19*, 255-265.

Van Hoof, D., Passier, R., Ward-Van Oostwaard, D., Pinkse, M.W., Heck, A.J., Mummery, C.L., and Krijgsveld, J. (2006). A quest for human and mouse embryonic stem cell-specific proteins. Mol Cell Proteomics *5*, 1261-1273.

Vastenhouw, N.L., Zhang, Y., Woods, I.G., Imam, F., Regev, A., Liu, X.S., Rinn, J., and Schier, A.F. (2010). Chromatin signature of embryonic pluripotency is established during genome activation. Nature *464*, 922-926.

Wu, Z., Irizarry, R.A., Gentleman, R., M., M.F., and F., S. (2004). A Model Based Background Adjustment for Oligonucleotide Expression Arrays. Johns Hopkins University, Dept of Biostatistics Working Papers *Working Paper 1*.

Xie, S.Q., Martin, S., Guillot, P.V., Bentley, D.L., and Pombo, A. (2006). Splicing speckles are not reservoirs of RNA polymerase II, but contain an inactive form, phosphorylated on serine2 residues of the C-terminal domain. Mol Biol Cell *17*, 1723-1733.
